# Topical treatment of diabetic foot ulcers using a novel quercetin-loaded hyaluosome gel nanoformulation

**DOI:** 10.1038/s41598-026-37537-4

**Published:** 2026-02-17

**Authors:** Mohamed S. Amer, Khalid A. El-Nesr, Fatma I. Abo El-Ela, Mohamed I. Zanaty

**Affiliations:** 1 Biotechnology and Life Sciences Department, Faculty of Postgraduate Studies for Advanced Sciences, Beni-Suef University, Beni-Suef, 62511 Egypt; 2 Pathology Department, Faculty of Veterinary Medicine, Beni-Suef University, Beni-Suef, 62511 Egypt; 3 Pharmacology Department, Faculty of Veterinary Medicine, Beni-Suef University, Beni-Suef, 62511 Egypt; 4 Biotechnology and Life Sciences Department, Faculty of Postgraduate Studies for Advanced Sciences, Beni-Suef, 62511 Egypt

**Keywords:** Diabetic foot ulcers, Quercetin, Hyaluosomes, Inflammatory cytokines, Antioxidants, TGF-β, Biochemistry, Biotechnology, Diseases, Drug discovery

## Abstract

Diabetic foot ulcers (DFUs) are among the most severe and debilitating complications of diabetes mellitus, often progressing to limb amputation due to persistent, non-healing lesions. Because of the multifactorial pathogenesis of DFUs, there is a pressing demand for novel drug delivery approaches capable of enhancing treatment effectiveness. This study aimed to enhance topical drug delivery for DFU management through the formulation and optimization of a Quercetin-loaded hyaluosome gel (QCN-HS). The nanoformulation was prepared and characterized, followed by in vitro and in vivo evaluations. The optimized QCN-HS demonstrated an entrapment efficiency of 88.1%, a mean particle size of 122.42 nm, and a zeta potential of − 24 mV. Morphological assessment by transmission electron microscopy revealed a uniform cuboidal-to-rounded square architecture without aggregation, further validated by FTIR spectroscopy. QCN-HS significantly reduced the expression of pro-inflammatory cytokines (TGF-β, TNF-α, IL-17, and IL-6) and lowered MPO activity, while markedly increasing GST and GSH levels. Moreover, QCN-HS downregulated ADAMTS-5 and MMP-13 while upregulating TIMP-3, findings corroborated by scratch assay and IC50 data, confirming effective regulation of extracellular matrix turnover. Histological and immunohistochemical analyses further demonstrated significant suppression of NF-κB expression in skin tissue. The quercetin-loaded hyaluosome gel, with favorable physicochemical properties and high entrapment efficiency, showed strong therapeutic potential for DFU treatment. Both in vitro and in vivo results underscore its ability to attenuate inflammation, enhance antioxidant defenses, and promote extracellular matrix remodeling; these outcomes strengthen the rationale for QCN-HS as an effective targeted treatment for DFUs.

## Background

Diabetes mellitus (DM) is a persistent and multifaceted metabolic disease characterized by chronic hyperglycemia arising from defects in insulin secretion, insulin action, or both^1^. It has emerged as a critical and rapidly growing public health concern, affecting an estimated 537 million individuals in 2022; this number is projected to rise to 643 million by 2030 and 783 million by 2045^2^. The disorder is accompanied by a spectrum of serious long-term complications that can profoundly impair quality of life and place an immense strain on healthcare resources. These complications are typically classified as microvascular, encompassing diabetic neuropathy, retinopathy, and nephropathy, or macrovascular, including peripheral arterial disease, stroke, and coronary artery disease^3^.

Among these sequelae, diabetic foot ulcers (DFUs) stand out as one of the most devastating and costly outcomes. Globally, DFUs affect an estimated 6.3–11% of the diabetic population, with a lifetime risk exceeding 25%^4–6^. They account for approximately one million lower-limb amputations each year, representing up to 85% of all diabetes-related amputations worldwide^7^. DFUs arise from a multifactorial pathophysiology in which peripheral neuropathy reduces protective sensation and peripheral arterial disease compromises blood flow and tissue integrity. This combination results in persistent, non-healing wounds highly susceptible to infection^5^. The growing prevalence of multidrug-resistant (MDR) pathogens further complicates clinical management, as the overuse and misuse of antibiotics accelerate antimicrobial resistance and limit available treatment options^8^. This scenario underscores the pressing need for novel, non-antibiotic therapies that can effectively combat infection, alleviate inflammation, and promote tissue repair.

Bioactive phytochemicals have gained increasing interest as complementary agents in wound care. Among these, Quercetin (3,3′,4′,5,7-pentahydroxyflavone; QCN) is a widely distributed plant-derived flavonoid found in apples, onions, tea, nuts, seeds, and numerous other botanical sources^9^. It exhibits potent antioxidant, anti-inflammatory, and antimicrobial properties^9^. Despite its therapeutic potential, the clinical use of QCN is hindered by poor water solubility, chemical instability, and limited dermal penetration, all of which result in low bioavailability.

Advances in nanotechnology-based delivery systems offer promising solutions to these limitations. Hyaluosomes, vesicular nanocarriers composed of phospholipids and cross-linked hyaluronic acid, exhibit excellent biocompatibility, enhance skin hydration, and enable deeper dermal penetration and sustained release of active compounds^10–12^. Hyaluronic acid (HA) significantly enhances Haylasome, an advanced delivery system that surpasses liposomes, niosomes, nanogels, and free HA, by optimizing hydration, moisturization, and permeability^10–12^. These previous characteristics position hyaluosomes as an attractive platform for improving the topical delivery and therapeutic efficacy of QCN.

In the present work, we designed and characterized a topical QCN-loaded hyaluosome hydrogel intending to enhance QCN solubility, stability, and skin permeability. We hypothesized that this nanoformulated system would accelerate wound closure, modulate inflammatory responses, and reduce microbial load in diabetic wounds. By combining QCN’s multifaceted bioactivity with the targeted delivery and controlled-release properties of hyaluosomes, this strategy aims to address persistent challenges in DFU therapy and contribute to the development of effective wound-healing interventions.

## Materials and techniques

### Material

QCN (Q4951-10G) had been acquired from the pharmaceutical company Sigma-Aldrich (Germany) and maintained at 4 °C until it was required. Chloroform and methanol were obtained from Supelco Analytical Products (Germany), while sodium deoxycholate was obtained from Loba Chemie Ltd (India). Streptozotocin (STZ) was acquired from Sigma-Aldrich (Germany). Cholesterol and lecithin were obtained from Alfa Aesar (UK). The Algomhoria Chemical Company (Egypt) provided each separate chemical and reagent, which was an analytical standard. Using colorimetric assay kits (SPINREACT™, S.A./S.A.U., Santa Coloma, Spain), we determined glucose and lipid profile markers, and LDL-C was calculated according to^13^ Eq. 1.

1 $$\:\boldsymbol{L}\boldsymbol{D}\boldsymbol{L}-\boldsymbol{C}\:=\:\boldsymbol{T}\boldsymbol{C}\:-\:\left[\boldsymbol{H}\boldsymbol{D}\boldsymbol{L}-\boldsymbol{C}\:+\:\frac{\boldsymbol{T}\boldsymbol{G}}{5}\right]$$

Glycated hemoglobin (HbA1c) and insulin concentrations were assessed using the ichroma™ fluorescence immunoassay analyzer (Boditech Med Inc. ™, Korea). ELISA kits from FINE TEST™ (Wuhan Fine Biological Technology Co., China) were employed to quantify inflammatory cytokines (IL-6 and IL-17), while an ELISA kit from Abcam™ Ltd. (UK) was employed to quantify TNF-α. Protein expression levels of MMP-13, ADAMTS-5, and TIMP-3 were evaluated by Western blotting using kits from Santa Cruz Biotechnology™ Inc. (USA). (GST), (GSH) and (MPO) were quantified using ELISA kits. The GST kit was purchased from Creative Diagnostics™ (USA), while the GSH and MPO kits were obtained from Bio Vision™ Inc. (Milpitas, USA). The RNA extraction was performed using the Direct-zol™ RNA Miniprep Plus kit (Cat# R2072) from ZYMO RESEARCH CORP. (USA), which is designed for high-quality RNA purification from samples in TRI Reagent^®^. For reverse transcription and PCR amplification, the SuperScript™ IV One-Step RT-PCR System (Cat# 12594100) from Thermo Fisher Scientific (USA) was utilized. This kit combines both cDNA synthesis and PCR amplification in a single reaction, featuring high thermostability and processivity with the inclusion of M-MuLV reverse transcriptase, Taq DNA polymerases, SYBR Green dye, and ROX dye for efficient detection and analysis of RNA.

### Preparation of quercetin-hyaluosomes nano hybrid gel (QCN-HS)

The preparation of QCN-HS gels by the thin-film hydration method^14^. Briefly, Quercetin (0.09 g) was dissolved in 5 ml methanol, and this solution was added to cholesterol (0.06 g), sodium deoxycholate (0.08 g), and lecithin (0.4 g) that was dissolved in 10 ml chloroform in a round-bottom flask and then evaporated until dry in a rotary evaporator (Rota vapor, Heidolph VV 2000, Burladingen, Germany) at 100 rpm for 30 min at 25 °C, forming a lipid thin film. The obtained film was hydrated with an aqueous solution of 0.012 g of hyaluronic acid (HA) in PBS, 6 ml; the pH is 7.4, for 60 min with stirring at room temperature. Then, ultrasonication (Ney ultrasonic cleaner, USA) was performed for 30 min to produce unilamellar vesicles and decrease their particle size. Subsequently, add 3% sodium carboxymethylcellulose (NACMC). After that, stir the mixture magnetically for approximately one minute to create a 2.5% Quercetin-hyaluosomes Nano Hybrid Gel.

### Characterization of Quercetin hyaluosomes

#### Determination of QCN-HS size and potential

Dynamic laser scattering was employed to assess the average size, polydispersity index, and zeta potential of QCN-HS using the Nanos ZS90 from Malvern’s Zetasizer Nano series (Malvern Instruments, UK). QCN-HS (50 µL) was dispersed in PBS (950 µL) at a dilution of 1:20 to obtain a total volume of 1 mL before every measurement. The measurements were taken at 25 °C. All measurements were performed in triplicate (*n* = 3)^15^.

#### Examination of transmission electron microscopy

The morphology and ultrastructural characteristics of the QCN-HS nanocarriers were investigated using transmission electron microscopy (TEM) with a Tecnai G2 20 S-TWIN instrument (FEI, Netherlands) at an accelerating voltage of 200 kV. A carbon-coated copper grid (400 mesh) was meticulously coated with a drop of the appropriately diluted QCN-HS vesicular dispersion, which was allowed to adhere for one minute. Filter paper was employed to meticulously eliminate the surplus liquid. The sample was air-dried at ambient temperature (~ 25 °C) for 10 min before imaging and was then negatively stained with 2% (w/v) phosphotungstic acid (PTA, pH ~ 6.8) to improve contrast. To assess the structural integrity, consistency, and morphology of the vesicles, micrographs were acquired at varying magnifications^16^.

#### Entrapment efficiency (EE %) determination

To assess the entrapment efficiency (EE%), the QCN-HS formula was centrifuged at 14,000 rpm for 45 min at 4 °C to remove (free) QCN from the vesicular fraction. The free QCN in the resulting supernatant was meticulously collected and analyzed using a UV–visible spectrophotometer (λ = 265 nm) in comparison to a Quercetin standard calibration curve. In order to guarantee reproducibility, each measurement was conducted in triplicate (*n* = 3). Equation No. 2, which was employed to determine the entrapment efficacy, was as follows:^17^.

$$\:EE\left(\%\right)\left[\frac{\left(\mathrm{T}\mathrm{o}\mathrm{t}\mathrm{a}\mathrm{l}\:\mathrm{a}\mathrm{m}\mathrm{o}\mathrm{u}\mathrm{n}\mathrm{t}\:\mathrm{o}\mathrm{f}\:\mathrm{Q}\mathrm{C}\mathrm{N}\:-\mathrm{F}\mathrm{r}\mathrm{e}\mathrm{e}\:\mathrm{a}\mathrm{m}\mathrm{o}\mathrm{u}\mathrm{n}\mathrm{t}\:\mathrm{o}\mathrm{f}\:\mathrm{Q}\mathrm{C}\mathrm{N}\right)}{\mathrm{T}\mathrm{o}\mathrm{t}\mathrm{a}\mathrm{l}\:\mathrm{a}\mathrm{m}\mathrm{o}\mathrm{u}\mathrm{n}\mathrm{t}\:\mathrm{o}\mathrm{f}\:\mathrm{Q}\mathrm{C}\mathrm{N}}\right]*100$$

#### Fourier transform infrared spectroscopy

To characterize the chemical interactions and surface adsorption of functional groups within the QCN-HS formulation and its constituent components, Fourier-transform infrared spectroscopy (FTIR) was implemented for QCN and QCN-HS. The FTIR analysis offers a potential perspective on molecular interactions, such as hydrogen bonding or van der Waals forces, that may affect the encapsulation and stability of drugs within nanocarriers^18^. Spectra were recorded using a Vertex 70 FTIR spectrophotometer (Bruker, Karlsruhe, Germany). For each sample, 1 mg of dry material (pure QCN, excipients, and QCN-HS formulation) was homogenized with 100 mg of potassium bromide (KBr) and compressed into transparent pellets using a hydraulic press under vacuum^19^. Spectral acquisition was performed over the wavenumber range of 400–4000 cm⁻¹, with a spectral resolution of 1 cm⁻¹ and an accumulation of 64 scans per sample. All measurements were conducted at a controlled ambient temperature of 25 °C^20^. The resulting spectra were analyzed for characteristic vibrational bands to assess functional group retention, drug-excipient compatibility, and possible molecular interactions between Quercetin and the hyaluosomes matrix.

#### In vitro release study QCN AND QCN-HS

Aliquots of QCN-HS (equivalent to 3 mg QCN) were placed in glass cylinders (2.5 cm × 6 cm) sealed with a dialysis membrane (MWCO 12,000 Da)^21^. The release medium consisted of 200 mL Sorensen’s phosphate buffer (pH 5.5) with 0.01% v/v Tween 80, maintained at 37 ± 0.5 °C under stirring at 200 rpm. At predetermined intervals (1, 2, 4, 6, 8, 16, and 24 h), 2 mL aliquots were withdrawn and replaced with fresh medium to maintain constant volume. Free QCN solution (1 mL, 3 mg/mL in PBS pH 7.4 with 60% v/v PEG 400) was also tested as a control. Release was monitored spectrophotometrically at λ = 265 nm, with all measurements performed in duplicate^22^.

### In vitro cytotoxicity assay and IC_50_ for QCN and QCN-HS

The MTT assay relies on the reduction of yellow tetrazole MTT (3-(4,5-dimethylthiazol-2-yl)-2,5-diphenyltetrazolium bromide) into purple formazan crystals by mitochondrial reductase enzymes in viable cells. The amount of formazan formed directly correlates with cellular metabolic activity and thus serves as a quantifiable indicator of cell viability^21^.

#### Cells and samples

##### Assessment of cytotoxicity on human cell lines for QCN and QCN-HS

Cell viability was determined through a colorimetric assay based on the mitochondrial-mediated bioreduction of the yellow tetrazolium salt MTT (3-(4,5-dimethylthiazol-2-yl)-2,5-diphenyl tetrazolium bromide) into insoluble purple formazan crystals. This transformation, driven by active mitochondrial dehydrogenases in living cells, serves as a reliable indicator of metabolic activity and cellular health^23^.

#### MTT assay for QCN and QCN-HS

Experiments were carried out aseptically in a Class II A2 Laminar Flow Cabinet (Labconco, USA). Human foreskin fibroblast (HSF) cells were cultured in DMEM with 1% antibiotic-antimycotic, 1% L-glutamine, and 5% fetal bovine serum at 37 °C/5% CO₂^24^. After 10 days, cells were harvested with trypsin-EDTA, counted, and seeded into 96-well plates at 1 × 10⁴ cells/well^25^. Following 4 h adherence, cells were exposed to graded drug concentrations (1,000–15.625 µg/mL) in triplicate, with untreated controls, for 24 h. At 48 h, MTT solution was added and incubated for 4 h at 37 °C, 5% CO₂, then formazan was solubilized using 10% sodium dodecyl sulphate (SDS) in 0.01 M HCL, and plates were incubated overnight^26^. Viability and cytotoxicity were calculated according to Equation No. 3^27^.

$$\:Viability\:=\:\left(\frac{absorbance\:of\:medication}{\:absorbance\:of\:control\:}\right)*\:100$$

$$\:Cytotoxicity\:=\:100\:-\:viability$$

#### Scratch wound healing assay for QCN and QCN-HS

The migratory capacity of human skin fibroblast (HSF) cells was evaluated using a wound scratch assay^28^. Briefly, HSF cells (5 × 10⁵/well) were inoculated into 6-well plates and cultured overnight at 37 °C with 5% CO₂ to establish confluent monolayers. A linear wound was created with a sterile 10 µL pipette tip, and wells were rinsed with PBS to remove debris. Cells were then treated with DMEM + 1% FBS containing test compounds at their IC50 concentrations. Wound closure was monitored at 0, 24, and 48 h using an inverted microscope with ZEISS ZEN (blue edition) imaging software. Migration was quantified by measuring wound width reduction relative to baseline.

### Characterization of the synthesized hydrogel formulation

#### PH measurement

The pH of QCN and QCN-HS hydrogel formulation was evaluated using a pre-calibrated digital pH meter. The electrode was gently inserted into direct contact with the hydrogel, and readings were recorded after a 1-minute stabilization period to ensure accuracy. This step is critical to confirm the hydrogel’s suitability for biological applications, as pH influences stability and biocompatibility^29^.

#### Spreadability (dispensability) evaluation

To assess the spreadability of QCN and QCN-HS hydrogel, approximately 0.50 ± 0.05 g of the sample was placed at the center of a watch glass with a 5 cm diameter. A second identical watch glass was carefully placed on top, and the system was left undisturbed for 5 min. The diameter of the resultant circular spread was measured and recorded. Spreadability is a crucial parameter for topical formulations, reflecting ease of application and surface coverage^29^.

#### Homogeneity

QCN and QCN-HS hydrogel samples were collected from multiple points within the batch to evaluate formulation homogeneity. Each sample (1 g) was dissolved in 9 mL of phosphate-buffered saline (PBS, pH 5.5) and subjected to UV–Vis spectrophotometric analysis to determine uniform distribution of the active constituents. This approach ensures consistency in drug delivery and performance across the hydrogel matrix^30^.

#### Rheological analysis

The rheological behavior of QCN and QCN-HS hydrogel formulations was assessed using a programmable cone-and-plate viscometer. A 0.5 g sample was applied to the lower plate, and measurements were taken at 25 ± 1 °C using a circulating water bath to maintain temperature consistency. A shear rate ranging from 20 to 400 s⁻¹ was applied using spindle 52 to simulate varying mechanical stresses during application and skin contact. This viscoelastic characterization is essential to predict the formulation’s mechanical performance and user acceptability^31^.

### In vivo study

#### Animals and experimental design

All animal procedures were approved by the Ethics Committee of the Faculty of Postgraduate Studies for Advanced Sciences, Beni-Suef University, Egypt (Approval No.: PSAS-BSU-HAREC.025/08/08), and conducted in accordance with institutional, national, and international animal care guidelines. Twenty-five healthy adult male albino rats (*Rattus norvegicus*, 140–160 g) were housed under controlled conditions (12:12 h light–dark cycle, 25 ± 2 °C, 50–60% humidity) in standard polypropylene cages (five per cage), with free access to water and a standard rodent diet on an ad libitum basis^32^. Type 2 diabetes mellitus (T2DM) was induced by feeding rats a high-fat, high-carbohydrate diet (HFHCD; 45% fat, 35% carbohydrates) for 21 days, followed by a single low intraperitoneal dose of streptozotocin (STZ, 10 mg/kg body weight) in 0.1 M sodium citrate buffer (pH 4.5)^33^. Blood glucose was measured 48 h post-STZ injection using a MINDRAY semi-auto chemistry analyzer, and animals with glucose > 200 mg/dL were classified as diabetic^34^. Rats were divided into five groups (*n* = 5 per group), and balanced for body weight and glycemic status before treatment. This study was conducted and is reported in accordance with the ARRIVE guidelines (Animal Research: Reporting of In Vivo Experiments).

#### Induction of diabetic foot wound

A standardized diabetic foot ulcer (DFU) model was created in rats under anesthesia induced by intraperitoneal ketamine–xylazine (9:1; ketamine 90 mg/kg, xylazine 5 mg/kg; 0.1 mL/100 g body weight)^35^. After confirming anesthesia, a full-thickness circular wound (6 mm) was made on the dorsal hind foot using a sterile biopsy punch and micro scissors, avoiding tendon and vascular injury^36,37^. Wounds were left untreated and uncovered to simulate chronic DFU conditions. Digital images were captured on days 0, 1, 4, 6, 9, and 13, and wound areas were quantified by pixel-based measurements using a ruler-calibrated reference scale^38–40^. The percentage of wound closure (%WC) was calculated according to Equation No. 4^41^.

$$\:{\%}\mathrm{W}\mathrm{C}=100\times\:\:\frac{intial\:wound\:area-wound\:area\:at\:{N}^{TH}\:DAY}{intial\:wound\:area\:}$$

#### Grouping of lab animals and starting topical drug loading

1. Group 1 (G1): Normal untreated rats (Control Negative).
2. Group 2 (G2): Diabetic Foot Ulcer (Control Positive).
3. Group 3 (G3): DFU treated with Quercetin gel 3%.
4. Group 4 (G4): DFU treated with Quercetin loaded on hyaluosomes gel 3%.
5. Group 5 (G5): DFU treated with Fucidin cream 2% (Standard Treatment).

Fucidin cream (fusidic acid) was selected as a standard treatment to reflect current clinical practice in chronic wound management, although its primary action is antibacterial rather than anti-inflammatory or antioxidant.

### Biochemical markers

#### Blood sampling

After the final clinical assessment, animals were fasted overnight and euthanized with an intraperitoneal injection of ketamine-xylazine (0.1 mL/100 g body weight). After deep anesthesia, the animals were euthanized by cervical dislocation. Blood was obtained from the jugular vein for biochemical analysis^42^. For serum preparation, blood was drawn into vacutainer gel tubes; for plasma glucose measurement, sodium fluoride tubes were used; and for HbA1c determination, EDTA tubes were employed. All samples were stored at – 20 °C until analysis.

#### Skin tissue sampling

Skin tissue inflammatory markers analyzed included cytokines (IL-6, IL-17, and TNF-α), matrix-degrading enzymes (ADAMTS-5 and MMP-13), and the matrix inhibitor TIMP-3. Following euthanasia, rat skin tissues were thoroughly rinsed with ice-cold water, blotted on filter paper, and weighed on an analytical balance^39^. Tissues were homogenized using a Polytron homogenizer at 40 °C to prepare a 10% homogenate in 0.05 M phosphate buffer (pH 7). The homogenate was centrifuged at 10.000 rpm for 20 min to remove cell debris, nuclei, erythrocytes, and mitochondria^40^. The supernatant (cytoplasmic extract) was used for marker detection: ELISA kits quantified IL-6, IL-17, and TNF-α, while Western blot kits measured MMP-13, ADAMTS-5, and TIMP-3. Band intensities were quantified by densitometric analysis using Image J and normalized to the housekeeping protein β-actin. Results are expressed as relative protein expression in arbitrary units (AU).

### Oxidative stress markers

Skin tissue oxidative markers analyzed included GST, GSH, and MPO. Approximately 50 to 100 mg of skin tissue was weighed and transferred to pre-chilled tubes. Ice-cold phosphate-buffered saline (PBS, pH 7.4) was incorporated at a 1:10 (w/v) ratio, and samples were homogenized thoroughly with a mechanical homogenizer on ice. The homogenates underwent centrifugation at 10.000 × g for 15 min at 4 °C to eliminate cell debris. The supernatants were collected and stored at – 80 °C for subsequent use in the ELISA assays^43^.

### Molecular investigations

#### Total RNA extraction and quantitative real-time PCR (qRT-PCR)

Total RNA was extracted from rat skin tissue using the Direct-zol RNA Miniprep Plus kit (ZYMO RESEARCH CORP.)^44^ According to the manufacturer’s instructions. Tissue was homogenized in TRI Reagent^®^, mixed with ethanol, and loaded onto a Zymo-Spin™ IIICG Column, followed by centrifugation. In-column DNase I treatment was applied to remove genomic DNA. RNA was washed, eluted, and quantified using a Beckman dual spectrophotometer by measuring OD at 260 nm. For gene expression analysis, the SuperScript™ IV One-Step RT-PCR kit (Thermo Fisher Scientific) with 2X Platinum™ SuperFi™ RT-PCR Master Mix was used^45^. The reaction mix included gene-specific primers for TGF-β and GAPDH (Table 1) and SYBR Green dye for fluorescence detection. Amplification was performed on a Step One Real-Time PCR system with the following profile: reverse transcription at 55 °C (10 min), denaturation at 95 °C (10 s), annealing at 55 °C (10 s), extension at 72 °C (30 s), and final extension at 72 °C (5 min). Relative quantification (RQ) of TGF-β expression was calculated using the ΔΔCt method, normalized to GAPDH^46^.

**Table 1:** Primer sequences used for quantitative real-time PCR.

Primer direction	Accession No.	Gene	Sequence (5’→3’)
Forward	NM_021578.2	*TGFB1*	GACTCTCCACCTGCAAGACC
Reverse	NM_021578.2	*TGFB1*	GGACTGGCGAGCCTTAGTTT
Forward	XM_017592435.1	*GAPDH*	CACCCTGTTGCTGTAGCCATATTC
Reverse	XM_017592435.1	*GAPDH*	GACATCAAGAAGGTGGTGAAGCAG

### Histopathological analysis

Full-thickness skin biopsies were collected from ulcer borders in both experimental and control groups and processed for histological evaluation with hematoxylin-eosin (H&E) staining^47^. Samples were fixed in 10% neutral buffered formalin, dehydrated through graded ethanol (70%, 90% for 1.5 h each; absolute ethanol for 3 h), and cleared with xylene for 4 h^48^. Paraffin infiltration was achieved using three sequential soft paraffin baths (1 h each), followed by embedding in pure paraffin wax at 58 °C^49^. Paraffin blocks were sectioned at 3–5 μm using a rotary microtome, mounted on slides, and stained with standard H&E protocol. After dehydration and xylene clearing, slides were permanently mounted with DPX. Microscopic examination was performed using a Leica DM500 at 40× magnification, and photomicrographs documented epidermal changes, dermal fibroblast density, vascularization, collagen organization, and inflammatory infiltrates across study groups^50^.

### Immunohistochemistry NF-κB examination

Immunohistochemistry (IHC) was performed using the avidin-biotin complex (ABC) method as previously described by Hsu et al.^51^. Paraffin-embedded tissue sections were deparaffinized in xylene and rehydrated through a graded ethanol series. Antigen retrieval was performed in a microwave oven for 5 min at 700 W using 0.01 M citrate buffer (pH 6.0), followed by cooling to room temperature. Sections were mounted in a Sequenza immunostaining system and incubated with peroxidase blocking solution for 10 min, then rinsed in PBS (pH 7.6). Next, they were treated with protein blocking serum for 10 min without rinsing. The primary antibody used was NF-kB p65/RelA rabbit polyclonal antibody (ABclonal Technology, USA; Cat#A2547; dilution 1:200), applied for 30 min at room temperature in a humid chamber. After rinsing in PBS, sections were incubated for 45 min with a biotinylated goat anti-rabbit secondary antibody (ABclonal Technology; Cat#AS014; dilution 1:10.000). This was followed by incubation with horseradish peroxidase-conjugated streptavidin for 20 min and visualization with 3,3′-diaminobenzidine (DAB) substrate for 5–10 min. Tissue sections were counterstained with Harris’s hematoxylin, passed through ascending grades of ethanol for dehydration, cleared in xylene, and permanently mounted with DPX.

### Statistical analysis

All statistical analyses were conducted with IBM SPSS Statistics v22.0 (IBM Corp., Armonk, NY, USA). Results are expressed as mean ± SEM. One-way ANOVA with Duncan’s post hoc test was used to assess group differences, with *p* < 0.05 indicating statistical significance.

## Results and discussion

### Characterization of quercetin hyalusomes nanoformula

#### Zeta potential and particle size analysis of QCN-HS

**Fig. 1:**
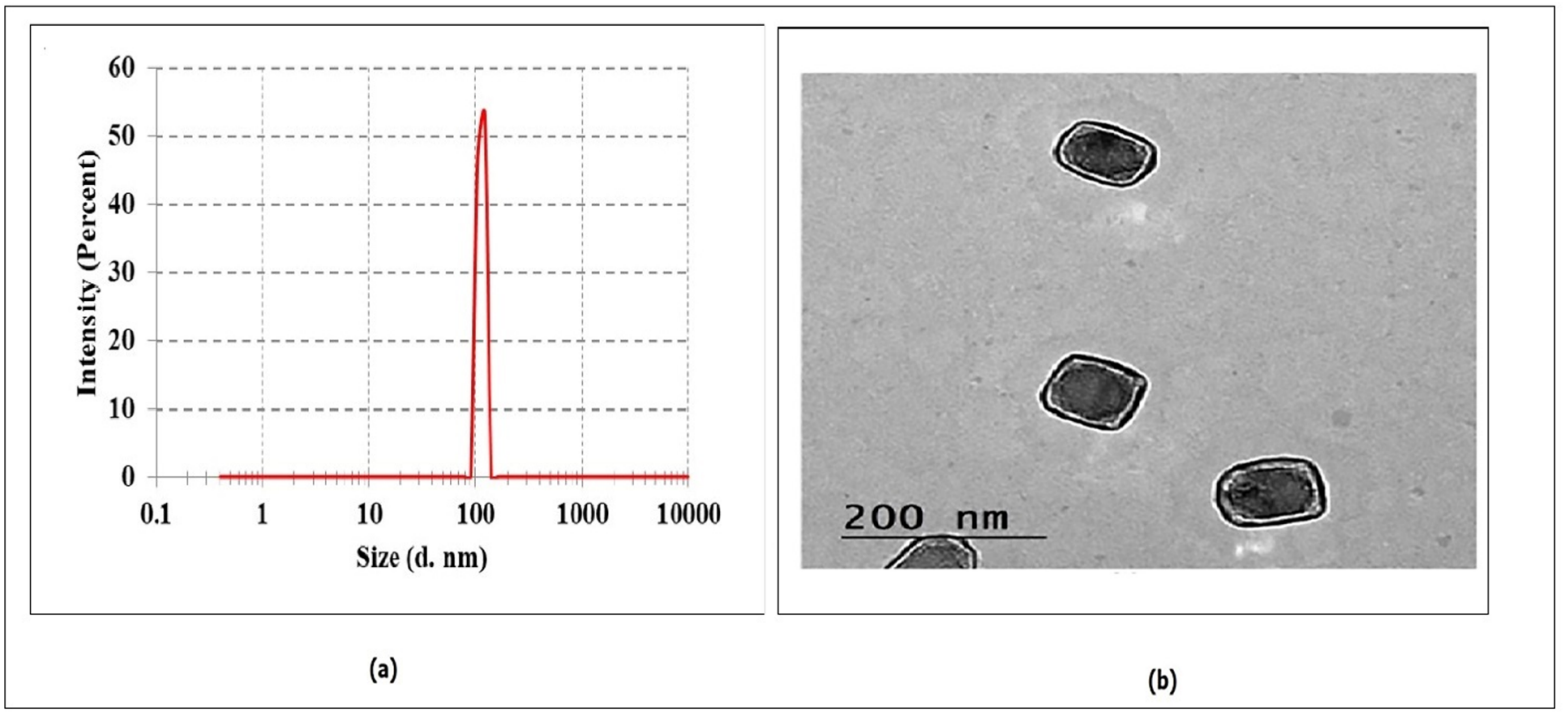
Characterization of QCN-HS showing (**a**) TEM micrograph of nanoparticles with rounded-square morphology and (**b**) size distribution by dynamic light scattering (DLS), indicating an average size of 122.42 nm.

Dynamic light scattering analysis indicated that the QCN-HS nanoformulation had an average hydrodynamic diameter of 122.42 nm (Fig. 1a), suitable for enhanced bioavailability and tissue penetration. The zeta potential was − 24 mV, attributed to the anionic nature of sodium deoxycholate (SDC) balanced with soy phosphatidylcholine (SPC), indicating excellent electrostatic stability and reduced aggregation. The polydispersity index (PDI) was 0.241, reflecting uniform particle distribution and high formulation quality (PDI < 0.3 indicates homogeneity). These results are consistent with Sharma et al.^52^, who reported similar colloidal stability in pH-sensitive quercetin nanocrystals. Collectively, the nanoscale size, surface charge, and PDI support the stability and biological compatibility of QCN-HS. Although the formulation demonstrated favorable physicochemical characteristics for topical use, the relatively wide particle size distribution could impact batch-to-batch reproducibility and biological efficacy. This underscores the necessity for additional optimization of the formulation in subsequent studies to enhance consistency and therapeutic performance.

#### Morphological insights into QCN-HS via TEM

Transmission electron microscopy (TEM) confirmed that QCN-HS nanoparticles were rounded-square in shape, with smooth edges, softened corners, and no visible aggregation (Fig. 1b). The measured size (~ 96 nm) was smaller than the hydrodynamic diameter from DLS, likely due to the absence of the hydrophilic shell in dry-state imaging. Such morphology is advantageous for enhanced cellular uptake and biodistribution. These findings are consistent with Wang et al.^53^, who reported that well-defined, smooth-surfaced nanoparticles without aggregation exhibit improved uptake and biodistribution. Moreover, the TEM and physicochemical data demonstrate the structural stability and suitability of QCN-HS for intracellular delivery and therapeutic applications.

#### Entrapment efficiency percentage (EE%) of QCN-HS

Hyalusome-based nanocarriers represent a promising platform for topical delivery of lipophilic bioactives, owing to their capacity to improve stability and facilitate skin penetration. The QCN-HS nanoformulation in this study showed a high loading efficiency of 88.1%. Quercetin, despite its strong antioxidant, anti-inflammatory, and antimicrobial activities^54^, suffers from poor solubility, low cutaneous absorption, and instability under physiological conditions^48,55,56^. Hyalusomes address these issues through their hyaluronic acid–rich structure, which improves dermal permeation, while their lipid bilayer and hydrogel matrix protect against oxidative, UV, and enzymatic degradation, as well as sustained release for prolonged skin residence^57^. Consistent with Maitra et al.^58^. Our results confirm that QCN-HS hydrogel enhances Quercetin stability, solubility, and dermal delivery, supporting its potential in reducing inflammation and promoting wound healing.

#### Fourier transform infrared spectroscopy reveals structural insights in QCN and QCN-HS

**Fig. 2:**
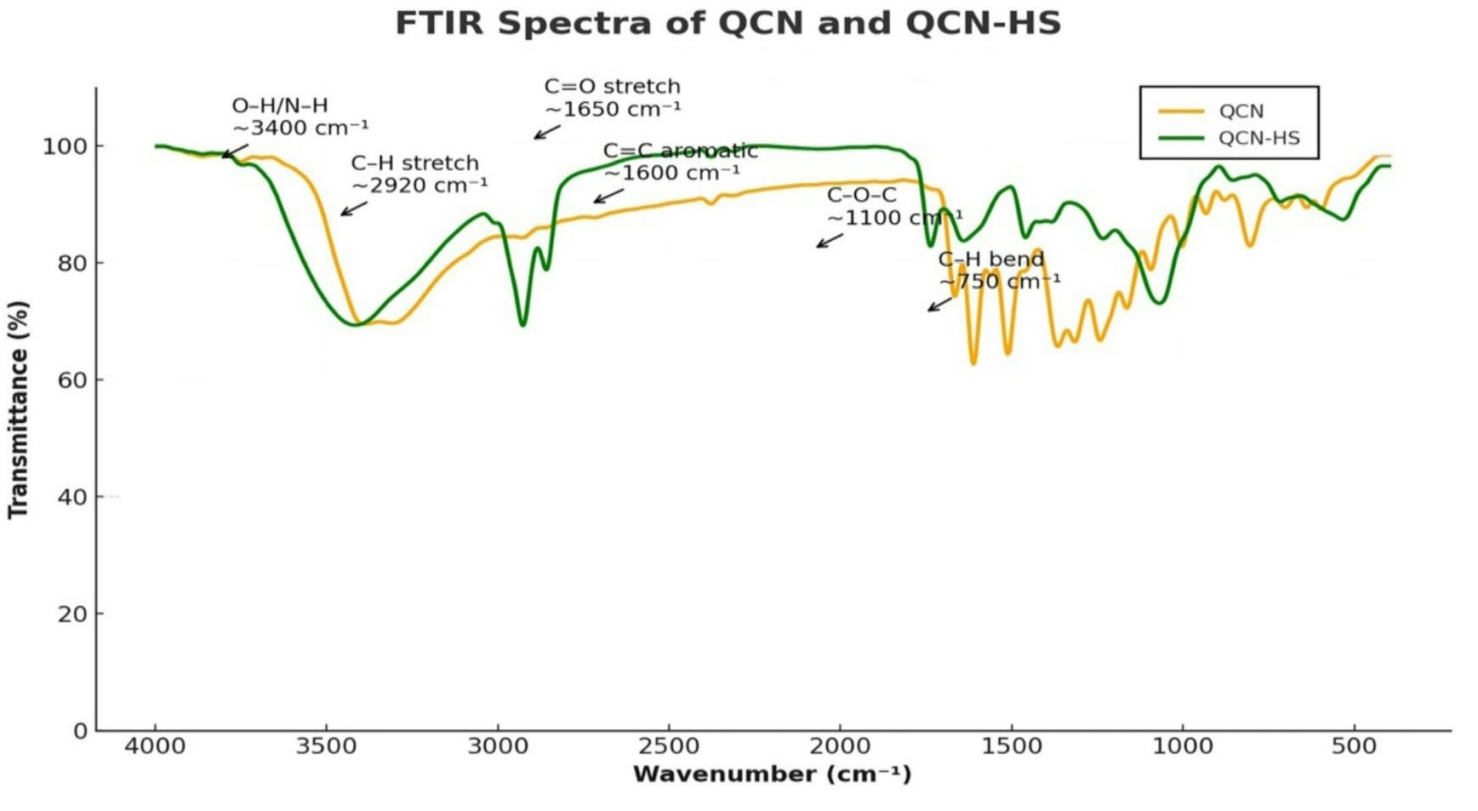
FTIR spectra of QCN and QCN-HS confirming successful quercetin incorporation into the hyaluosome nanocarrier.

Figure 2, FTIR analysis was conducted to characterize functional groups of quercetin (QCN) and QCN-HS. Pure QCN showed characteristic absorption bands, including a broad O–H stretch at 3410–3420 cm⁻¹, C = O stretching at 1660–1680 cm⁻¹, aromatic C = C stretches at 1600, 1510, and 1450 cm⁻¹, and C–O–C/C–OH vibrations between 1200 and 1000 cm⁻¹, confirming its polyphenolic structure. In QCN-HS, the O–H band (~ 3416 cm⁻¹) was broader and less intense, reflecting hydrogen bonding with hyaluronic acid and other excipients. Intensified C–H stretching peaks (2926, 2854 cm⁻¹) indicated lipophilic groups from phospholipids, while the C = O peak shift from 1660 cm⁻¹ to 1723 cm⁻¹ suggested interactions with the hyaluosome matrix. Additional peaks (1231, 1056, 731 cm⁻¹) corresponded to hyaluronic acid and lecithin components. Overall, the spectra of QCN and QCN-HS showed similar features with minor shifts, confirming successful encapsulation of QCN without covalent bonding to hyalusome components. These findings align with Nurjis et al.^59^ and previous nanoliposome encapsulation reports, supporting that QCN is stabilized within the hyalusome architecture via hydrogen bonding and electrostatic interactions.

### In vitro release dynamics of QCN versus QCN-HS

**Fig. 3:**
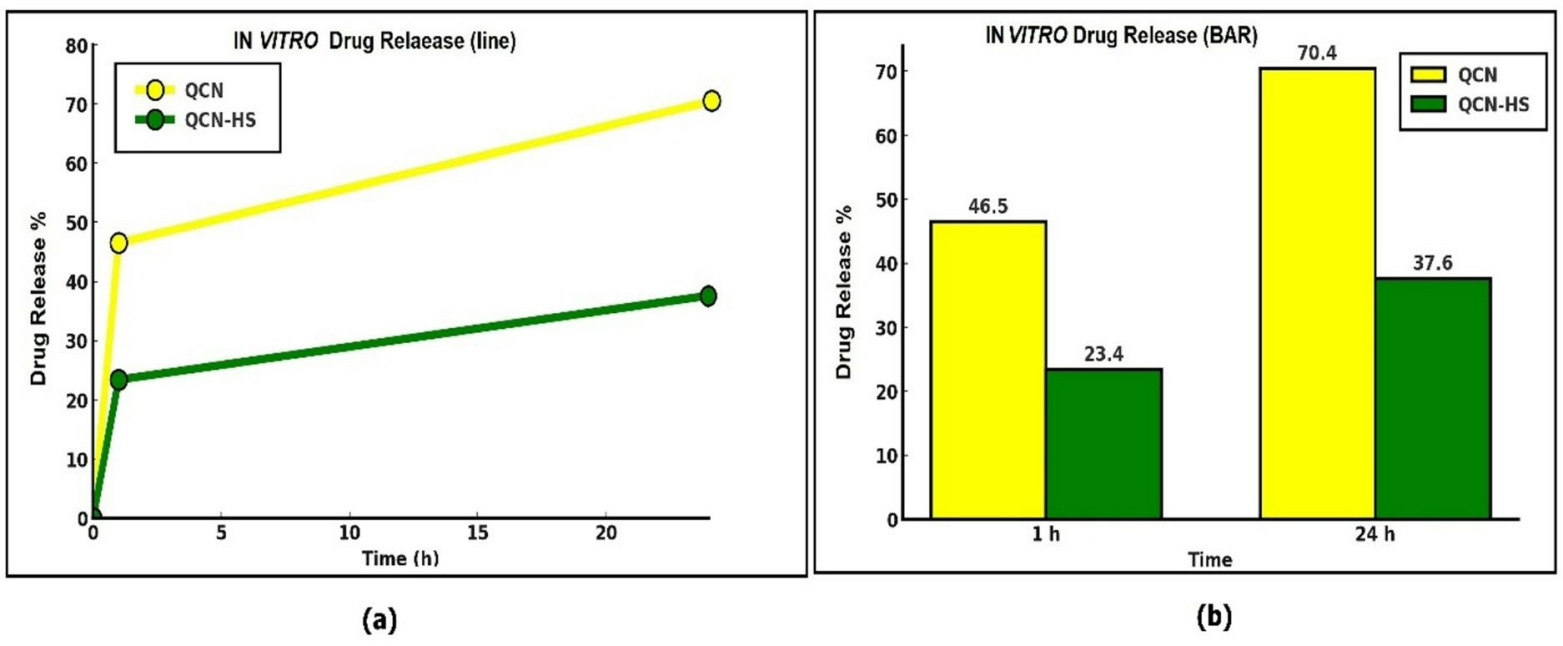
Drug release profiles of QCN and QCN-HS over 24 h: (**a**) cumulative release curves; (**b**) bar chart at 1 and 24 h of release.

Drug release studies (Fig. 3) revealed distinct differences between free QCN and QCN-HS over 24 h. Free QCN exhibited a rapid burst release of 46.5% within the first hour, reaching 70.4% by 24 h, reflecting quick dissolution and limited sustained activity. In contrast, QCN-HS released only 23.4% at 1 h and 37.6% at 24 h, demonstrating slower, controlled release with reduced burst effect, indicative of effective encapsulation within the hyalusome matrix. Such sustained release supports prolonged therapeutic availability. The data obtained align with Sivakumar et al.^60^, who reported controlled release with microsphere-based carriers. Confirming that QCN-HS provides enhanced stability and extended drug delivery.

### Comparative in vitro cytotoxicity evaluation of QCN and QCN-HS using MTT assay

**Fig. 4:**
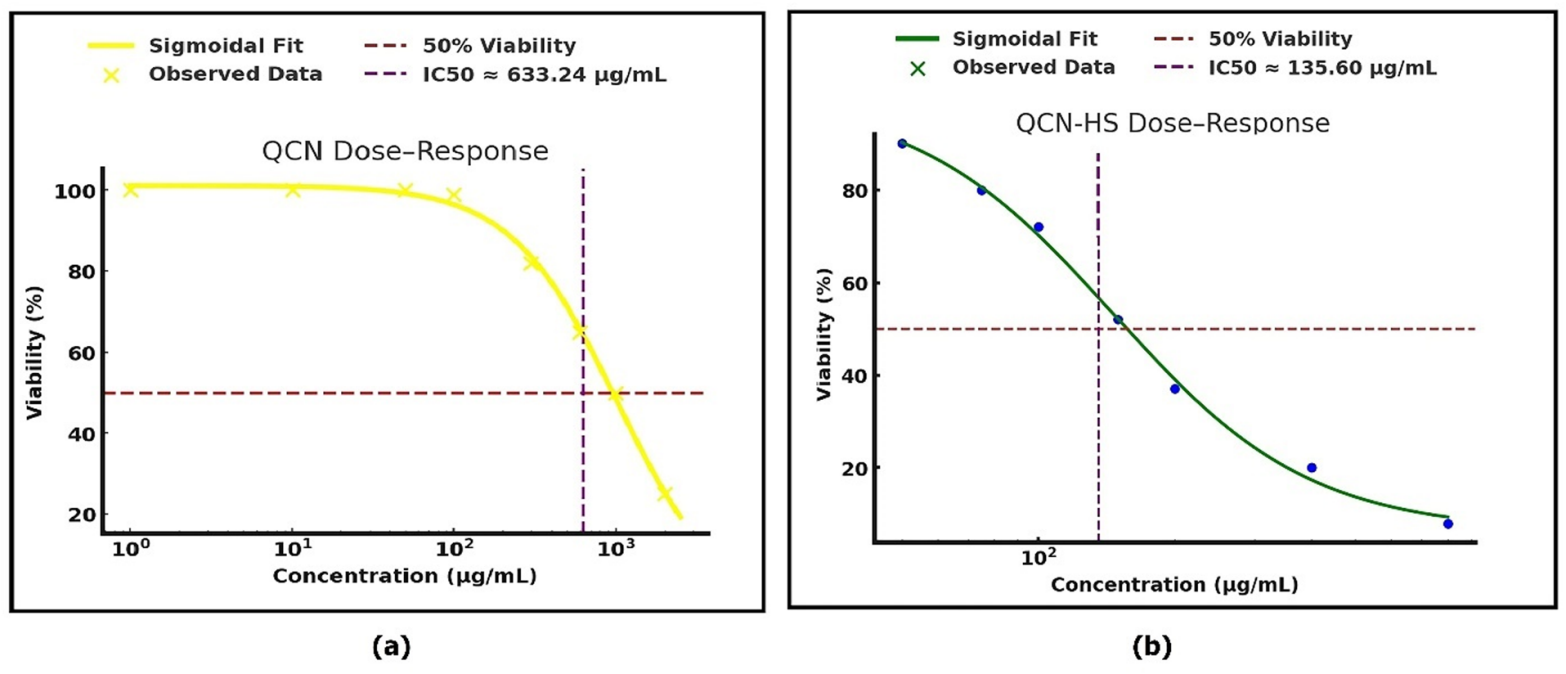
Cytotoxicity of QCN and QCN-HS by MTT assay: (**a**) QCN with IC50 633.24 µg/mL; (**b**) QCN-HS with IC50 135.60 µg/mL.

Cytotoxicity analysis (Fig. 4) revealed that QCN-HS displayed a sigmoidal dose–response. Where QCN-HS showed improved wound healing efficacy even at low doses, with an IC50 of 135.60 µg/mL compared to 633.24 µg/mL for free QCN. This may be explained by enhanced cellular uptake, penetration, and sustained release. The formulation also provides antifibrotic benefits, helping to prevent excessive fibroblast proliferation and scar formation. Notably, the administered dose of 12.5 mg per application (2.5% w/w) remains safely below the IC50, preserving fibroblast viability. The enhanced efficacy of QCN-HS aligns with reports of nanovesicle-based systems improving flavonoid delivery and retention, and contrasts with the limitations of inorganic antimicrobials such as silver^61,62^. QCN-HS normalized inflammatory and oxidative markers and restored skin integrity in vivo. Collectively, these findings confirm QCN-HS as a potent and safer nanoplatform for wound healing.

### In vitro characterization of QCN-HS hydrogel formulation

#### PH measurement

**Fig. 5:**
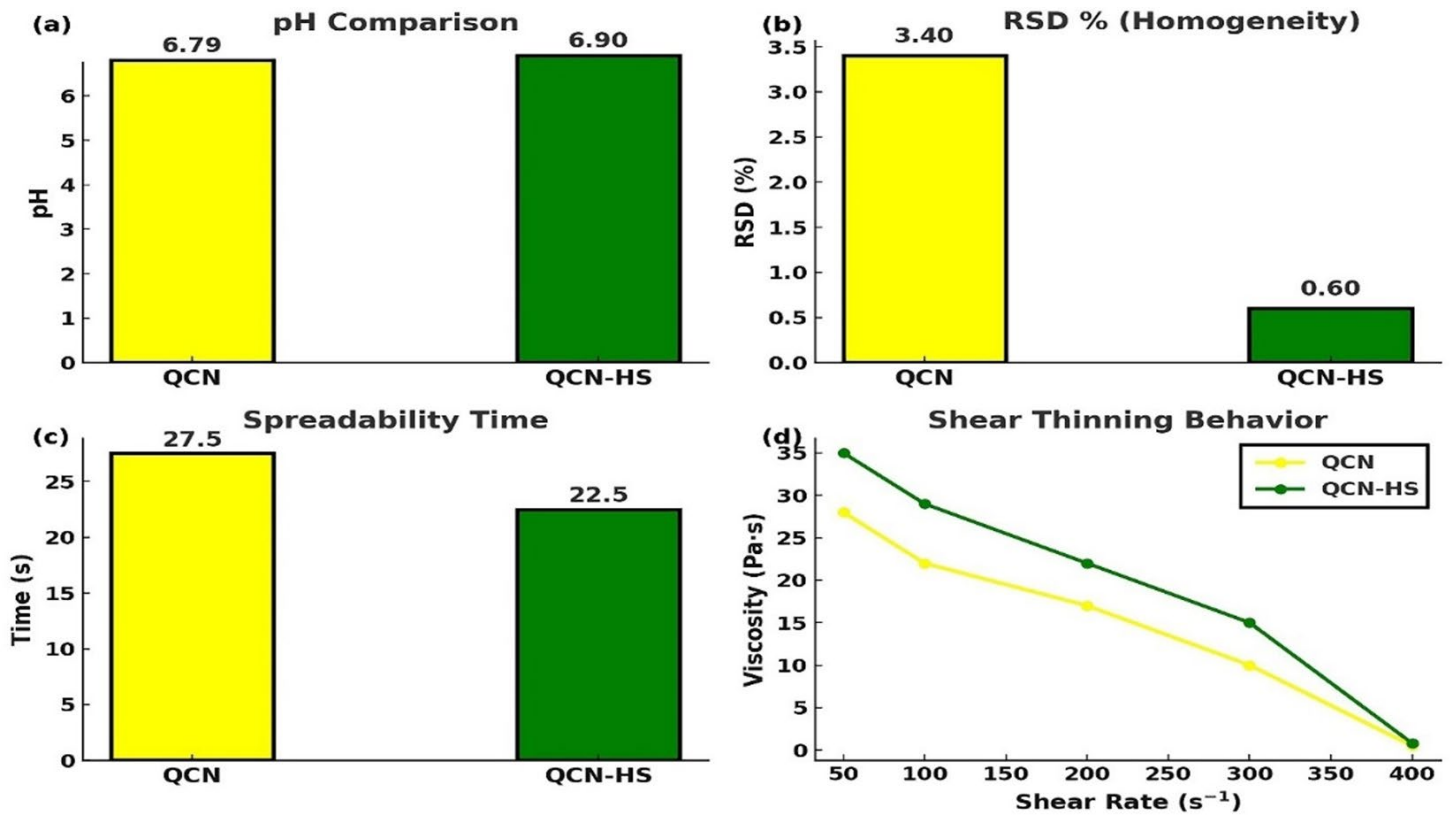
Physicochemical and rheological comparison of QCN and QCN-HS: (**a**) pH, (**b**) RSD% %, (**c**) spreadability, (**d**) rheological profile.

Figure 5a showed that QCN-HS hydrogel had a pH of 6.9, mildly acidic and within the physiologically acceptable range, attributed to contributions from QCN and hyaluronic acid (HA)^63^. By comparison, QCN gel exhibited a pH of 6.7 but, lacking HA vesicles, was more prone to environmental fluctuations. The pH profile of QCN-HS is advantageous, as mildly acidic formulations are known to minimize skin irritation and maintain barrier compatibility^56^.

#### Homogeneity and uniformity

Figure 5b, UV-Vis spectrophotometry at 265 nm confirmed uniform QCN distribution in QCN-HS hydrogel, with concentrations of 0.596–0.604 mg/mL (mean 0.6006 mg/mL) and a low relative standard deviation (RSD) of 0.60%. In contrast, the free QCN gel showed poorer uniformity (mean 0.545 mg/mL, RSD 2–5%) due to solubility issues and aggregation, increasing risks of crystallization, inconsistent dosing, and reduced stability. The low RSD of QCN-HS indicates superior compositional homogeneity, supporting reproducible dosing and therapeutic efficacy, consistent with previous findings^64^.

#### Spreadability and stability

Figure 5c showed that QCN-HS hydrogel maintained its structural and rheological integrity over 7 days, with viscosity increasing slightly from 17.18 to 17.55 Pa·s without affecting handling or application. Spreadability testing indicated smooth texture and ease of application, requiring 22–23 s to expand uniformly to a 3.5–4.0 cm radius. By contrast, the free QCN gel exhibited longer spreadability times (25–30 s) with smaller expansion (2.9–3.2 cm), along with less consistent viscosity due to aggregation and sedimentation. These findings are consistent with previous reports that nanostructured QCN integration enhances stability, uniform spreading, and formulation suitability^65^.

#### Rheological behavior and shear responsiveness

Rheological analysis from Fig. 5d showed that QCN-HS hydrogel exhibited pronounced shear-thinning, decreasing from ~ 35 Pa·s at 20 s⁻¹ to ~ 0.8 Pa·s at 400 s⁻¹. This behavior reflects hydrogen bonding and supramolecular entanglement among QCN, hyaluronic acid, and matrix components, providing stability at rest and reversible disassembly under stress to support spreadability and recovery. In contrast, the free QCN gel showed weaker shear-thinning (28 Pa·s → 1.5 Pa·s), lacking supramolecular organization and thus being more prone to phase separation. These findings align with prior reports that flavonoid nanostructure integration enhances stress-responsiveness, mechanical adaptability, and suitability for wound healing applications^66–68^.

### Enhanced in vivo diabetic wound healing mediated by QCN-HS compared to QCN

**Fig. 6:**
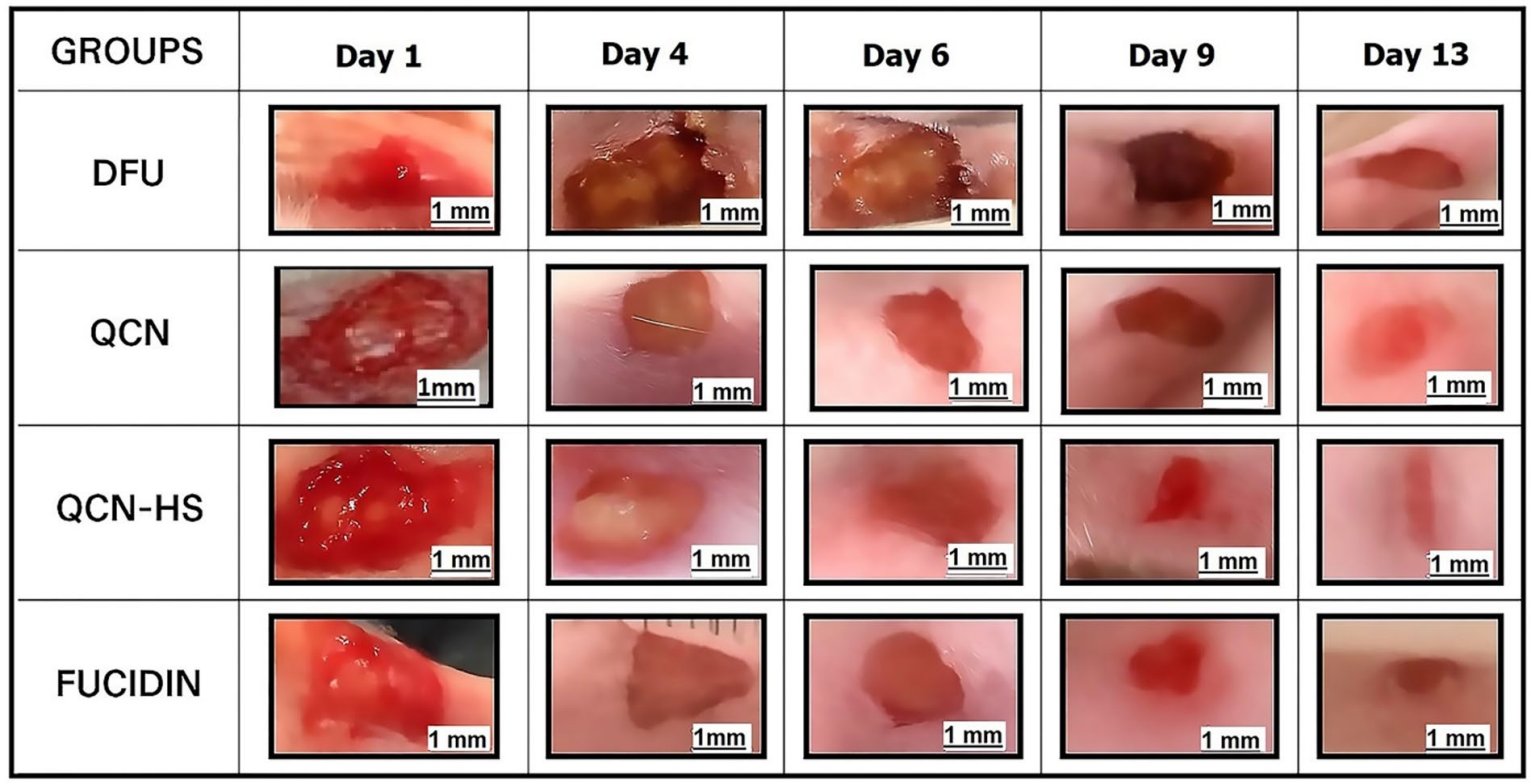
Photographic assessment of QCN and QCN-HS effects on diabetic foot ulcer healing.

**Fig. 7:**
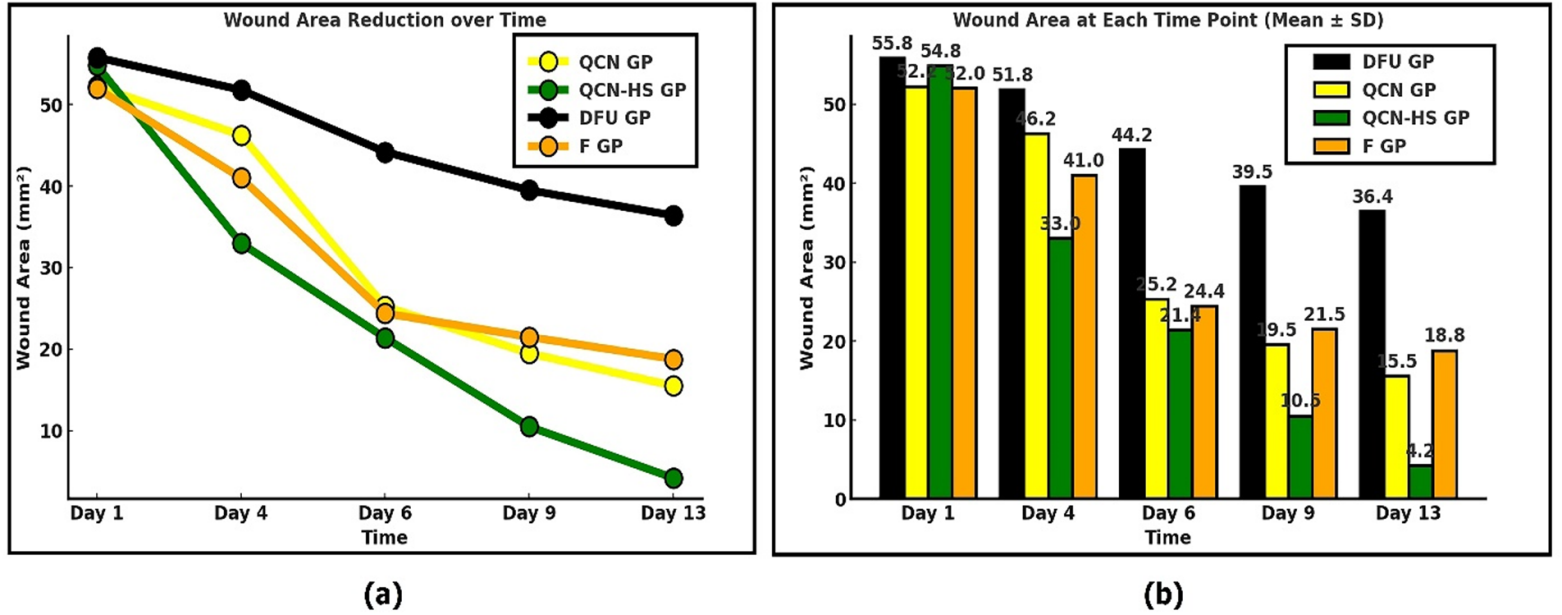
Wound closure progression in DFU models: (**a**) wound area reduction over time; (**b**) wound size measurements at specific days (mean ± SD).

The present study assessed the wound-healing effectiveness of different treatment formulations within different treated groups: QCN-GP, QCN-HS GP, and F-GP, compared to the diabetic foot ulcer (DFU) control group over 13 days. Fusidic acid is an effective treatment for Diabetic Foot Ulcers (DFU) due to its activity against Gram-positive bacteria, especially MRSA. It is used topically, providing localized infection control and reducing systemic side effects. Fusidic acid also offers anti-inflammatory benefits, promotes wound healing, and has a lower risk of resistance compared to other antibiotics. Its safety profile supports long-term use in chronic DFU management^69^. The data indicate a notable time-dependent decrease in wound size among all treated groups, with QCN-HS GP exhibiting the most significant therapeutic effect. Figures 6 and 7 showed that on day 1, all groups demonstrated similar initial wound sizes in millimeters, with no significant differences noted among DFU GP (55.80 ± 2.08), QCN-GP (52.25 ± 1.89), and F-GP (52.00 ± 1.22 mm). The QCN-HS GP group (54.80 ± 2.42) exhibited a statistically significant increase compared to DFU-GP and other treatment groups, indicating a consistent baseline across the groups. On Day 4, wound closure in the QCN-HS GP group (33.00 ± 2.53) demonstrated a significant reduction compared to the DFU GP (51.80 ± 2.27) and other treatment groups (QCN-GP: 46.25 ± 3.20; F-GP: 41.00 ± 1.41). On Day 6, the wound size in the QCN-HS GP group exhibited a significant reduction (21.40 ± 2.99), demonstrating superiority compared to all other groups (DFU GP: 44.20 ± 1.74; QCN-GP: 25.25 ± 1.55; F-GP: 24.40 ± 1.44). The differences were statistically significant, thereby confirming the sustained efficacy of QCN-HS gel. By Day 9, the downward trend in wound size continued, with QCN-HS GP showing a pronounced reduction (10.50 ± 1.20), markedly lower than QCN-GP (19.50 ± 1.40), F-GP (21.50 ± 1.10), and DFU GP (39.50 ± 1.50). On Day 13, the QCN-HS GP group demonstrated the most significant reduction in wound area (4.20 ± 0.80), surpassing both QCN-GP (15.50 ± 1.32) and F-GP (18.80 ± 0.97), and proving significantly more effective than the untreated DFU GP (36.40 ± 1.44). The QCN-HS GP treatment demonstrated consistently superior wound healing outcomes across all evaluated time points. The findings indicated that the QCN-HS delivery system offers an improved therapeutic effect, likely attributable to enhanced cellular uptake, prolonged release, and synergistic anti-inflammatory and antioxidant mechanisms. The findings support the efficacy of QCN-HS gel as a viable topical treatment for managing diabetic wounds. This aligns with recent studies on the role of flavonoids treated with nanoparticles in the wound healing of DFU disease^70,71^. In contrast to Chowdhury et al. (2024), who highlighted the promise of flavonoid nanoformulations but offered limited time-point detail, comparators, and mechanistic validation, our study offers concrete, repeatable proof of QCN-HS GP’s continuous advantage over all other formulations examined in diabetic wound healing. As a very promising targeted therapeutic, QCN-HS GP hastened closure, improved collagen organization, promoted angiogenesis, and decreased inflammation across a variety of experimental types. When compared to the study by Munusamy et al. (2025), which achieved 85% closure by Day 16 (90% with antibiotics), the QCN-HS GP system in the DFU model reached ~ 96% closure once daily for day 13, demonstrating both a faster healing trajectory and a superior outcome.

### Enhanced cellular migration in DFU models: comparative in vitro scratch assay of QCN-HS and QCN

**Fig. 8:**
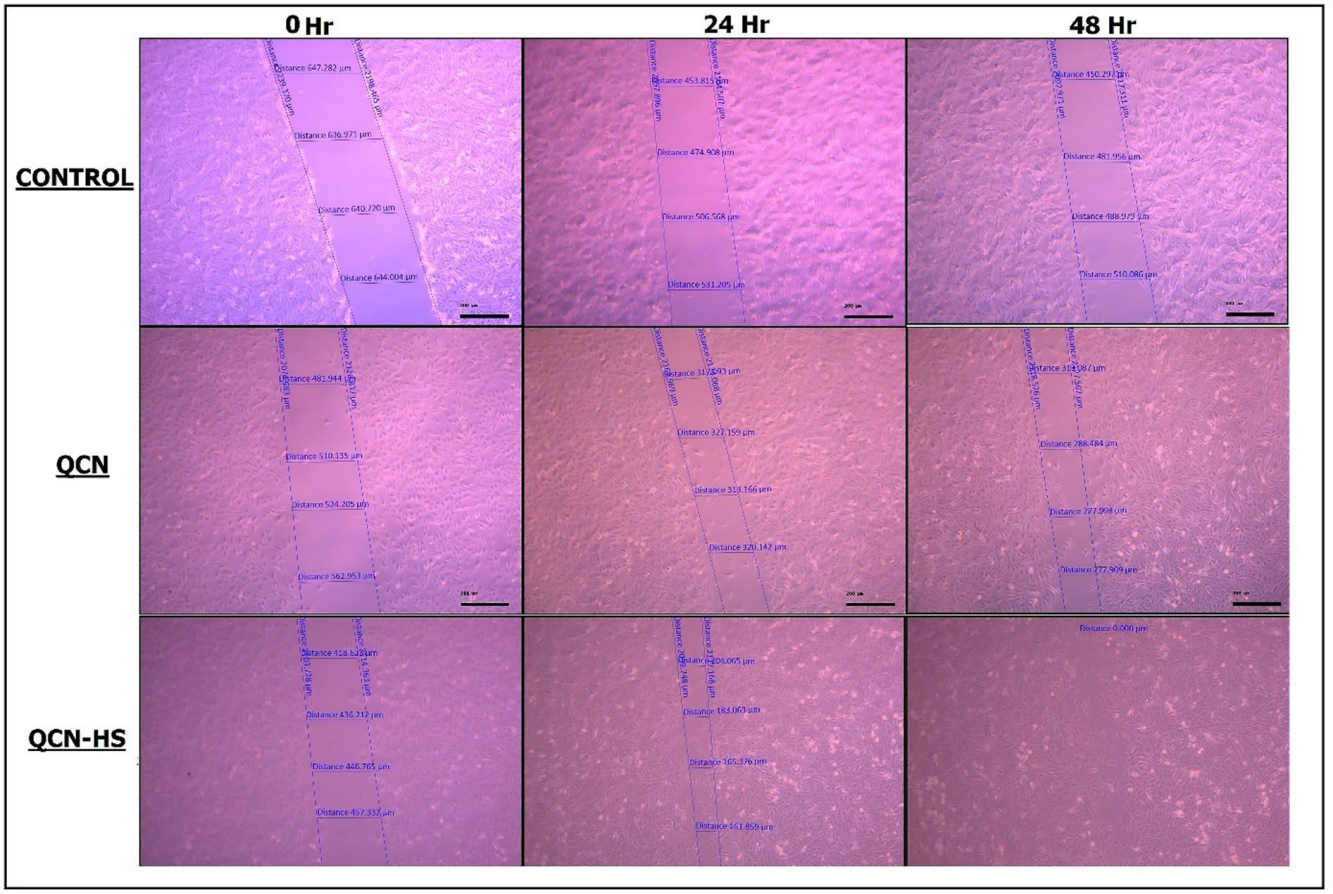
The photos of wound migration cells for the effect of QCN and QCN–HS after 0, 24, and 48 h.

**Fig. 9:**
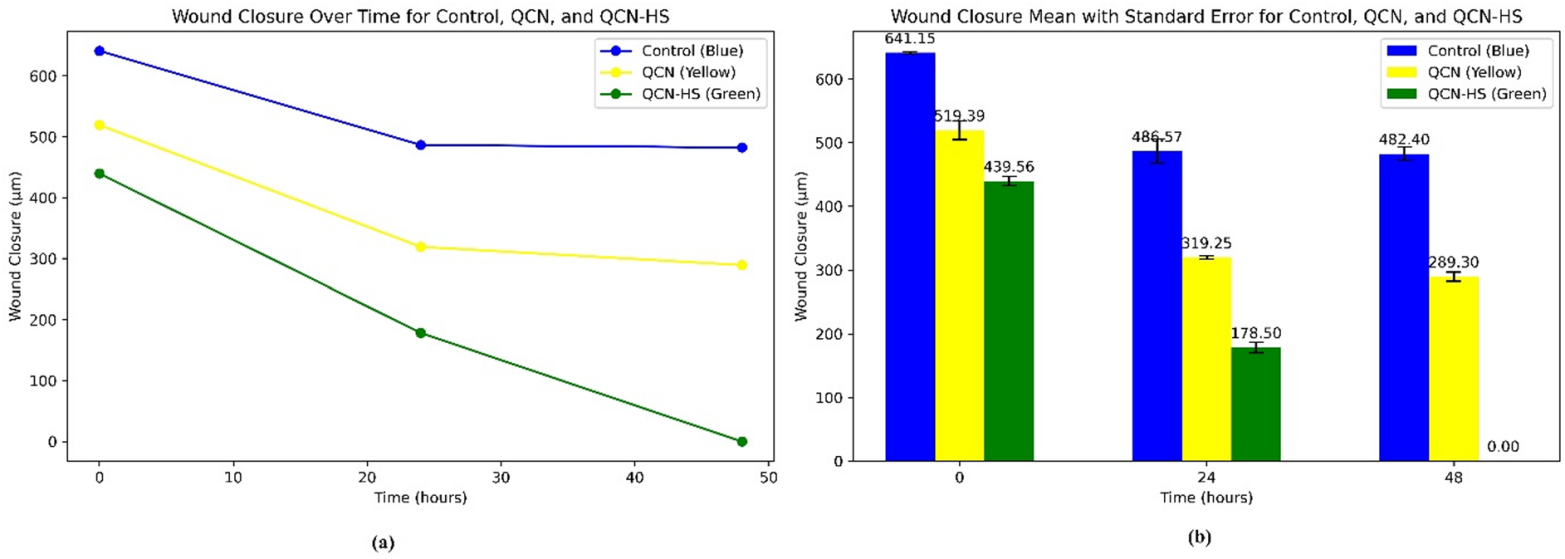
Scratch assay wound healing: (**a**) wound gap reduction over 48 h; (**b**) bar chart of wound width at 0, 24, and 48 h.

Cell migration plays a pivotal role in tissue regeneration and wound repair. In this study (Figs. 8 and 9, and Table 2), we evaluated the migration potential of human skin fibroblasts (HSFs) treated with quercetin (QCN) and its nano-hyaluosome formulation (QCN-HS), compared with untreated controls. The results revealed a time-dependent enhancement of cell motility, with QCN-HS markedly accelerating fibroblast migration relative to QCN alone and control. At baseline (0 h), no significant differences were observed among groups (Control: 641.15 ± 1.54 μm; QCN: 519.39 ± 14.6 μm; QCN-HS: 439.56 ± 7.2 μm), confirming initial homogeneity. By 24 h, a notable reduction in wound gap was exhibited in both QCN-HS and QCN groups, with QCN-HS demonstrating superior efficacy (178.51 ± 8.3 μm) compared to QCN (319.25 ± 2.5 μm) and control (486.57 ± 18.81 μm). At 48 h, this effect was further amplified: QCN-HS achieved complete wound closure (0.0 ± 0.0 μm), significantly outperforming QCN (289.3 ± 7.17 μm) and control (482.40 ± 10.73 μm). These findings suggest a potent enhancement of fibroblast migration mediated by the nanoformulation, likely attributable to improved cellular uptake and sustained bioavailability of QCN via the hyaluosome nanocarrier. Our results corroborate previous evidence that nano-encapsulation enhances QCN biological activity by protecting it from degradation and improving dermal tissue penetration^71^. Furthermore, the observed enhancement in migration is consistent with the known antioxidative and anti-inflammatory properties of QCN. Collectively, these data position QCN-HS as a promising candidate for promoting dermal repair in wound healing applications. Additional mechanistic studies and in vivo validation are warranted to elucidate the molecular pathways and translational potential of this nanoformulated therapy. These results are consistent with earlier reports highlighting the synergistic potential of nanoparticle-based drug delivery in regenerative medicine^72,73^. While van Rijt. (2017) primarily addressed the regenerative promise of nanoparticle-mediated delivery at a conceptual level^72^, our study provides unequivocal, empirically derived evidence that QCN-HS confers a marked kinetic advantage in fibroblast motility and gap closure dynamics, surpassing the performance of its non-encapsulated counterpart. While Kusnadi et al. (2024) outlined the wound-healing potential of collagen-based nanoparticles from a broad therapeutic perspective^73^, our study provides direct, quantitative evidence that QCN-HS achieves better fibroblast migration and gap-closure kinetics, surpassing the results reported for similar nanoparticle systems and clearly outperforming its non-encapsulated form.

**Table 2:** Wound closure (µm and %) over time for Control, QCN, and QCN-HS with standard.

Time	Control (µm)	QCN (µm)	QCN-HS (µm)	% Wound closure (Control)	% Wound Closure (QCN)	% Wound closure (QCN-HS)
0	641.15 ± 1.64	519.39 ± 14.6	439.56 ± 7.21	0%	0%	0%
24	486.57 ± 18.81	319.25 ± 2.5 1	178.5 ± 8.3 2	24.11%	38.5%	59.3%
48	482.40 ± 10.73	289.3 ± 7.17	0.0 ± 0.0	24.7%	44.3%	100%

### Biochemical markers

#### Lipid profile and validation of hyperlipidemia-induced diabetic phenotype before QCN-HS intervention

As shown in Table 3, the DFU-GP group exhibited markedly elevated serum total cholesterol, triglycerides, low-density lipoprotein cholesterol, and low HDL-C compared to NC-GP, confirming that all diabetic groups shared a uniform dyslipidemic state before intervention^74^. Pathophysiologically, diet-induced hyperlipidemia plays a central role in T2DM by promoting lipid accumulation in peripheral tissues, leading to systemic insulin resistance, β-cell dysfunction, and sustained hyperglycemia^75^. This lipotoxic situation further disrupts β-cell activity, impairs insulin signaling, and triggers oxidative stress and inflammatory cascades, which are recognized hallmarks of diabetes progression^76^. These results align with prior studies indicating that hyperlipidemic rodent models commonly develop diabetes through the interplay of insulin resistance, β-cell impairment, and chronic hyperglycemia^77,78^. The observed metabolic alterations in our DFU-GP cohort reinforce both the robustness and translational relevance of the experimental model, while establishing a compromised systemic baseline that ensures any subsequent improvements in wound healing following QCN-HS topical nanogel treatment can be attributed to local therapeutic action rather than systemic metabolic correction.

**Table 3:** Lipid profile parameters in all groups.

Parameters/groups	Cholesterol (mg/dl)	Triglycerides (mg/dl)	LDL (mg/dl)	HDL (mg/dl)
NC-GP	110.20 ± 2.13^c^	94.00 ± 2.42^b^	55.00 ± 1.41^c^	36.40 ± 1.20^a^
DFU GP	234.20 ± 2.95^a^	170.80 ± 7.87^a^	171.64 ± 4.69^a^	28.40 ± 0.67^b^
QCN - GP	230.00 ± 1.97^a^	170.60 ± 3.62^a^	170.08 ± 2.83^a^	25.80 ± 1.56^b^
QCN-HS GP	221.80 ± 2.10^b^	161.20 ± 1.20^a^	156.36 ± 2.68^b^	33.20 ± 0.96^a^
F- GP	227.40 ± 2.08^b^	168.00 ± 2.54^a^	169.32 ± 3.15^a^	24.40 ± 1.435^c^
P-value	0.000	0.000	0.000	0.0001

Data are expressed as mean ± SE, number of rats in each group (*n* = 5), means which share the same superscript symbol(s) are not significantly different, *P* < 0. 001.

#### Glycemic biomarker assessment to confirm diabetic status in the DFU model before QCN-HS treatment

As shown in Table 4, the DFU-GP group exhibited significantly higher fasting glucose and HbA1c levels, along with lower fasting insulin and increased HOMA-IR compared with non-diabetic controls, confirming a state of hyperglycemia and insulin resistance. Importantly, no glycemic differences were detected among the treated groups (QCN-GP, QCN-HS-GP, F-GP), demonstrating that all diabetic cohorts shared a comparable baseline metabolic dysfunction before topical therapy. This outcome reflects the diabetogenic synergy of the HFD–low-dose STZ model, where diet-induced insulin resistance is compounded by partial β-cell impairment caused by low-dose STZ (10 mg/kg body weight), thereby reproducing the dual defects characteristic of T2DM^79^. The data obtained align with prior evidence, validating the HFD–STZ protocol as a reliable model of sustained hyperglycemia and insulin resistance^80^, strengthening the translational value of our design and ensuring that any subsequent wound-healing benefits result from local therapeutic mechanisms rather than systemic glycemic changes.

**Table 4:** Glycemic index between all groups.

Parameters/groups	Fasting Insulin (ng/ml)	HOMA IR (U)	Fasting Blood Glucose (mg/dl)	HBA1c (%)
NC-GP	0.58 ± 0.37^a^	3.82 ± 2.4^a^	92.40 ± 2.58^c^	3.96 ± 0.067^b^
DFU GP	0.16 ± 0.02^b^	2.46 ± 0.3^b^	215.40 ± 3.64 ^b^	6.14 ± 0.06^a^
QCN –GP	0.14 ± 0.02^b^	2.30 ± 0.32^b^	230.20 ± 3.18^a^	6.06 ± 0.05^a^
QCN-HS-GP	0.16 ± 0.02^b^	2.63 ± 0.30^b^	230.80 ± 1.82^a^	6.06 ± 0.06^a^
F-GP	0.14 ± 0.02^b^	2.35 ± 0.33^b^	235.40 ± 1.83^a^	6.26 ± 0.13^a^
P-value	0.000	0.0001	0.000	0.0001

Data are expressed as mean ± SE, number of rats in each group (*n* = 5), means which share the same superscript symbol(s) are not significantly different, *P* < 0. 001.

### Effect of QCN-HS on tissue markers

#### Modulation of inflammatory cytokines by QCN-HS

**Fig. 10:**
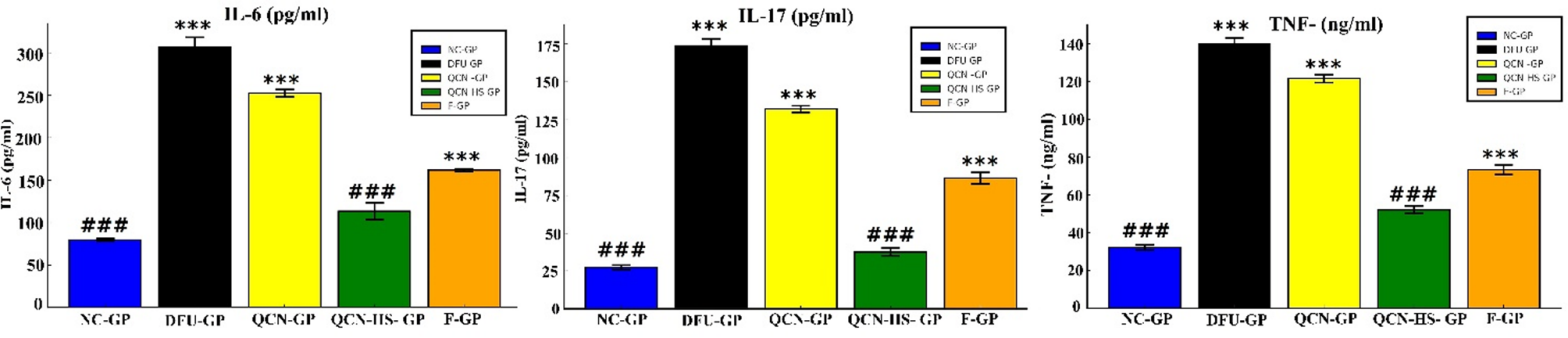
Effect of QCN and QCN-HS on inflammatory markers (IL-6, IL-17, and TNF-α) in different experimental groups. Bar charts represent mean ± SEM (*n* = 5). ***p < 0.001 vs. NC-GP; ^###^*p* < 0.001 vs. DFU-GP.

From Fig. 10, the DFU-GP group showed significantly elevated IL-6, IL-17, and TNF-α compared with the normal control (NC-GP), confirming that chronic inflammation is central to DFU pathogenesis. Where IL-6 persists in chronic wounds, causing tissue damage^81^ and IL-17 drives neutrophil infiltration and prolonged inflammation^82^. Furthermore, TNF-α inhibits collagen formation and angiogenesis, thereby impairing healing^83^.

Although the role of QCN as an anti-inflammatory agent was evident, it failed to significantly reduce cytokine levels compared with the DFU group. While QCN has well-established anti-inflammatory and antioxidant properties, its poor solubility and limited bioavailability restrict vascular penetration and limit sustained therapeutic efficacy^54,84^. In contrast, QCN-HS produced a marked decrease in IL-6, IL-17, in addition to TNF-α, approaching NC levels. The pronounced reduction in IL-6, IL-17, and TNF-α by QCN-HS is primarily attributable to enhanced intracellular delivery of QCN to key immune and endothelial cells at the wound site, facilitated by the hyaluosome nanocarrier. As demonstrated by Qiu S et al. (2024), sodium deoxycholate enhances vesicle penetration through inflammatory tissue, while the HA coating binds CD44 receptors to promote endocytosis into endothelial cells and immune cells^85^. The nano-sized (~ 122 nm) particles ensure deep tissue infiltration, reaching areas inaccessible to QCN. Finally, the gel matrix provides sustained local release, maintaining effective drug concentrations over time, which continuously suppresses pro-inflammatory cytokine signaling without the rebound effect seen with conventional formulations. This interpretation is consistent with the enhanced pharmacological performance achieved through nanocarrier-based delivery systems, which improve solubility, stability, tissue penetration, and sustained drug release^86,87^. These findings are consistent with previous reports on nanostructured flavonoids^88,89^.

#### Regulation of matrix remodeling enzymes and TIMP-3 by QCN-HS

**Fig. 11:**
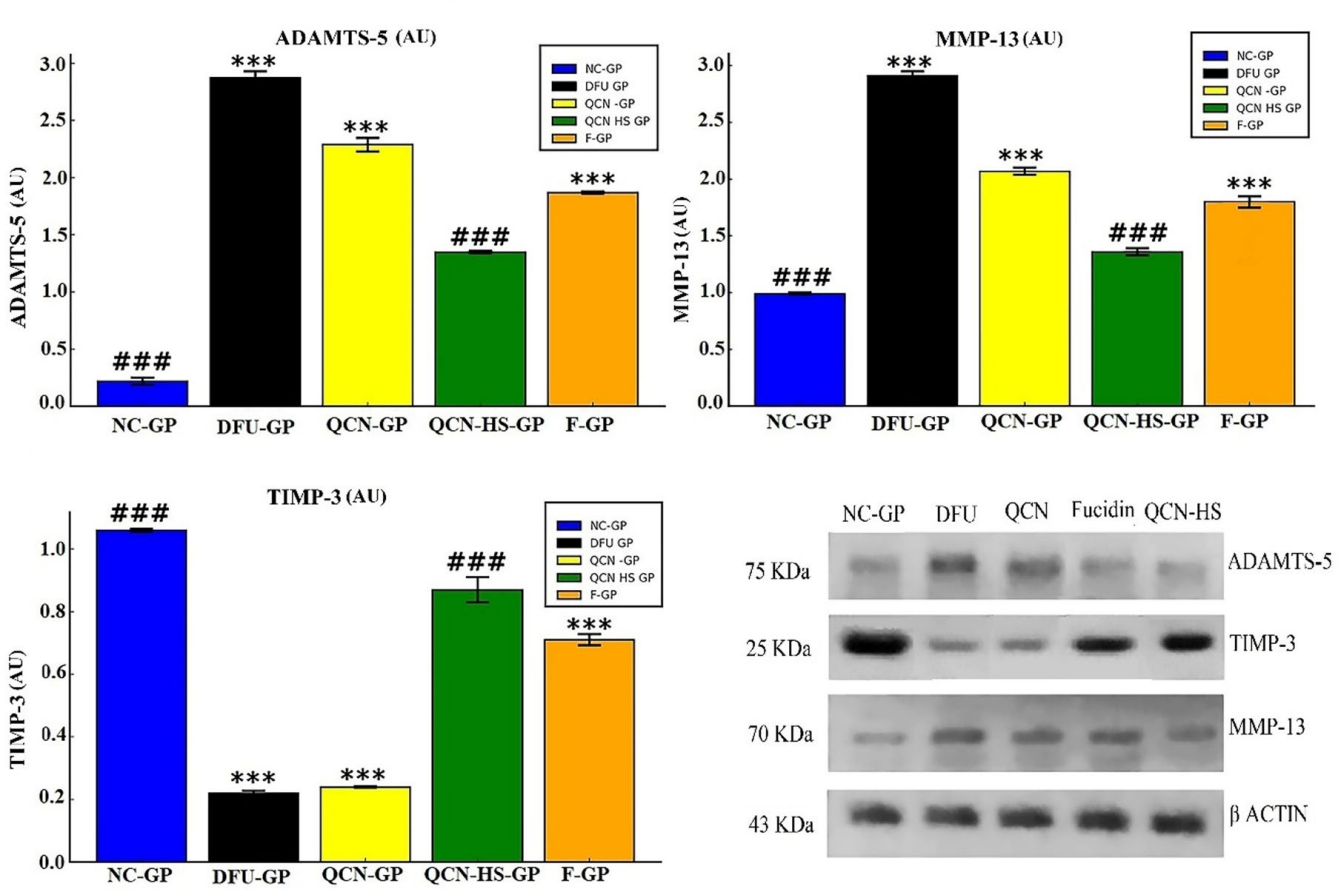
Effect of QCN and QCN-HS on ECM remodeling markers (ADAMTS-5, MMP-13, and TIMP-3) in different experimental groups. Protein expression levels were quantified by Western blot densitometry, normalized to β-actin, and expressed as relative protein expression (arbitrary units, AU). Bar charts represent mean ± SEM (*n* = 5). ***p < 0.001 vs. NC-GP; ^###^*p* < 0.001 vs. DFU-GP.

From Fig. 11, the DFU group (DFU-GP) exhibited significantly elevated levels of matrix remodeling enzymes (MMP-13 and ADAMTS-5) and reduced levels of tissue inhibitor TIMP-3 compared to the normal control (NC-GP). This imbalance reflects a disrupted MMP/TIMP axis, contributing to excessive ECM degradation and delayed wound healing in DFUs. TIMP-3 typically stabilizes the extracellular matrix (ECM) by inhibiting MMPs, ADAMs, and ADAMTS^90^. The elevated MMP-13 and ADAMTS-5, coupled with reduced TIMP-3, result in poor tissue remodeling, hampering re-epithelialization and angiogenesis^91,92^. In contrast, QCN-HS exhibited a significant reduction in MMP-13 and ADAMTS-5 while restoring TIMP-3 to its normal levels, demonstrating superior modulation of the ECM. QCN possesses notable anti-inflammatory and MMP-inhibitory properties; however, its therapeutic potential is hindered by low solubility and low bioavailability, which restrict its tissue penetration and capacity to effectively regulate ECM remodeling^93^. Meanwhile, the nanoformulation-enhanced membrane interaction could influence ECM remodeling more directly, optimizing the MMP/TIMP balance, as shown by Taherkhani et al. (2025)^94^.

### Modulation of oxidative stress markers by QCN-HS in DFU models

**Fig. 12:**
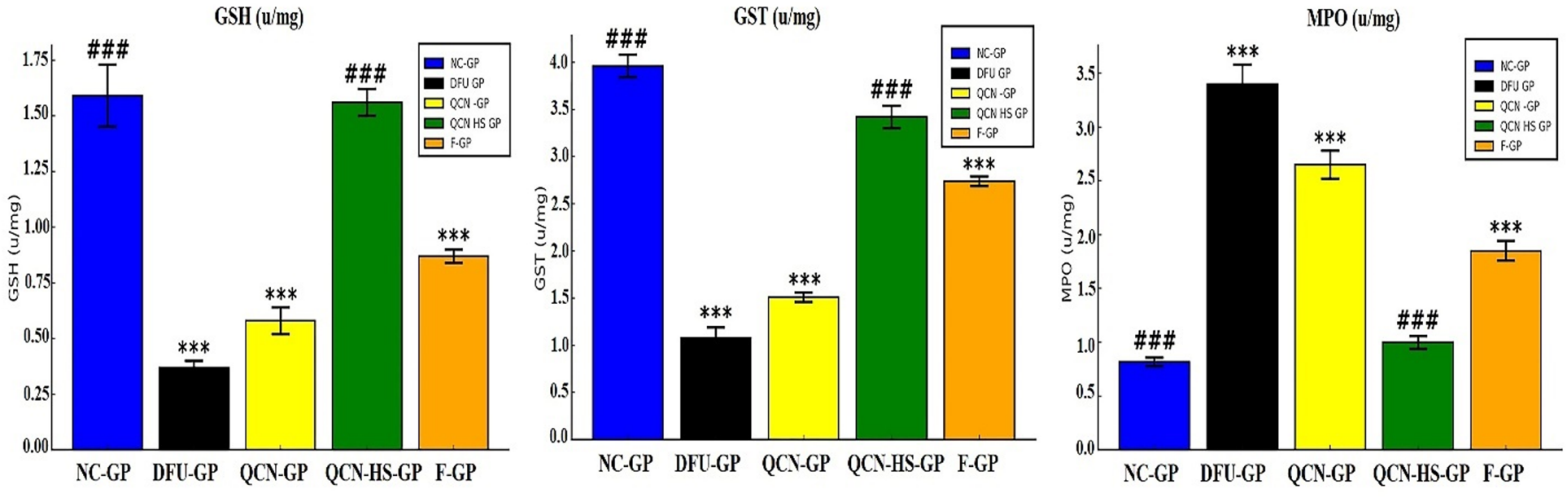
Effect of QCN and QCN-HS on Oxidative Stress (GSH, GST, and MPO) in different experimental groups. Bar charts represent mean ± SEM (*n* = 5). ***p < 0.001 vs. NC-GP; ^###^*p* < 0.001 vs. DFU-GP.

From Fig. 12, oxidative stress and antioxidant capacity were evaluated by measuring GST, GSH, and myeloperoxidase (MPO). DFU-GP displayed a significant decrease in GST and GSH levels alongside marked elevation in MPO levels compared to NC-GP, reflecting severe oxidative stress and impaired antioxidant defenses. DFUs are a severe complication of diabetes marked by chronic inflammation, impaired neovascularization, and delayed wound healing. The pathogenesis is driven by excessive production of reactive oxygen species, resulting in oxidative tissue damage and impaired cellular repair. Biochemical markers like GST, GSH, and MPO regulate oxidative balance and inflammatory responses during wound healing^95^. QCN-HS significantly restores GST and GSH to their normal levels while reducing MPO activity. QCN-HS potentiates endogenous antioxidant mechanisms, elevating GST activity and replenishing GSH levels. The nanoscale size of QCN enables deep tissue and cellular infiltration, thereby enhancing its ability to provide robust antioxidant and anti-inflammatory protection that is comparable to non-diabetic conditions, as demonstrated by Zhao et al. (2025)^96^. Previous study have reported that flavonoid-based nanoformulations, including QCN, can partially augment antioxidant enzymes (GST, GSH) and reduce pro-oxidative MPO in DFU models^97^.

### Regulatory impact of QCN-HS on gene expression in diabetic wound healing

**Fig. 13:**
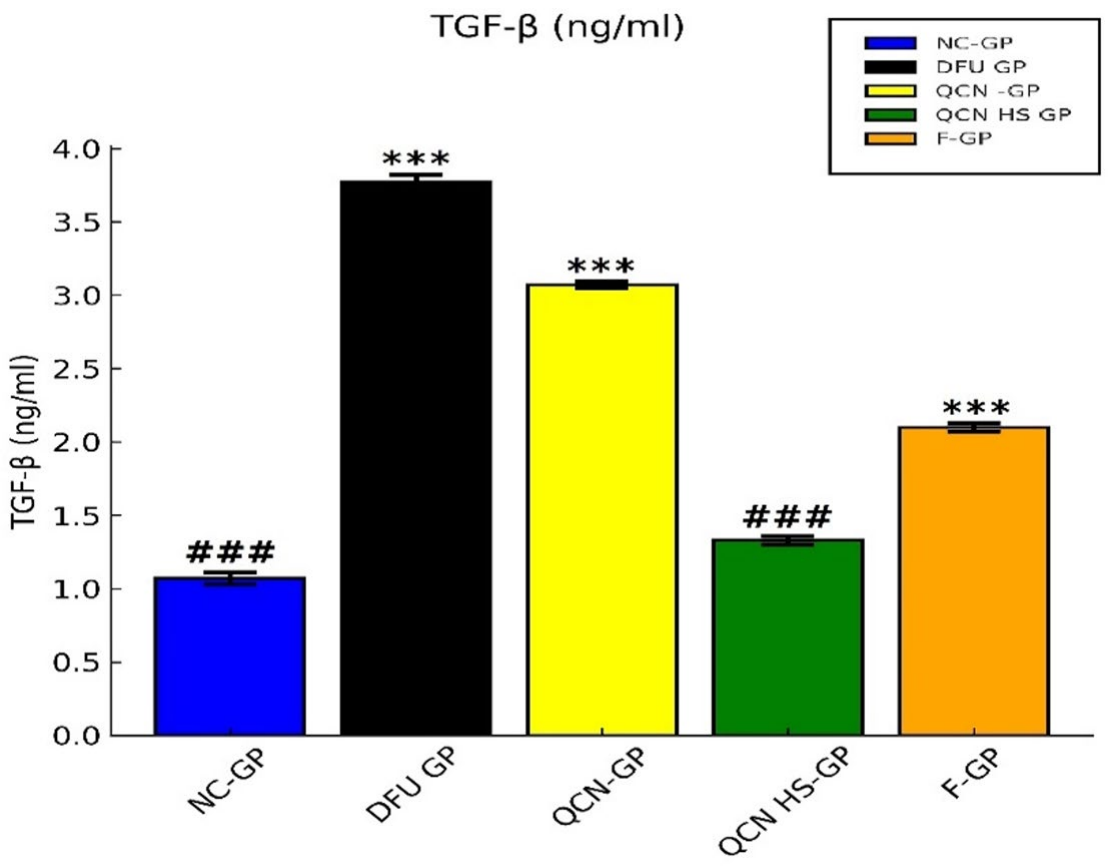
Effect of QCN and QCN-HS on tissue markers TGF β in different experimental groups. Bar charts represent mean ± SEM (*n* = 5). ***p < 0.001 vs. NC-GP; ^###^*p* < 0.001 vs. DFU-GP.

From Fig. 13, in our study, we applied quantitative real-time PCR (qPCR) to assess the amounts of Transforming Growth Factor-beta (TGF-β) mRNA in skin samples from several experimental groups. The TGF-β gene increased significantly in the DFU-GP relative to the NC-GP. TGF-β levels are elevated in diabetic foot ulcers (DFU), indicating a disrupted wound-healing response. TGF-β regulates inflammation, fibroblast activation, collagen deposition, and angiogenesis, but its sustained overexpression can lead to excessive extracellular matrix accumulation, fibrosis, and hindered remodeling, postponing granulation tissue development and wound healing. Persistent activity also extends the inflammatory phase and disrupts tissue regeneration and scarring, worsening DFU’s chronicity and non-healing characteristics^98^. In the present study, the TGF-β level was significantly reduced in QCN-HS-GP compared with DFU-GP. Quercetin modulates TGF-β signaling by attenuating its overactivation, thereby reducing fibrosis and inflammation in diabetic wounds. However, its therapeutic effectiveness remains limited in clinical use^99^. The hyaluosome-based nanocarrier of QCN-HS allows for localized QCN delivery directly into the wound, effectively restoring TGF-β levels to physiological ranges. By enhancing QCN solubility and promoting deeper tissue diffusion, the system achieves a controlled release that targets both endothelial and fibroblast compartments essential for TGF-β signaling. The resultant sustained intracellular QCN gradient stimulates TGF-β-dependent pathways, driving fibroblast expansion, extracellular matrix deposition, and angiogenesis. In parallel, TGF-β rebalancing fine-tunes the local inflammatory area and re-establishes tissue remodeling homeostasis, resulting in a coordinated acceleration of wound closure as shown by Rathana et al. 2024^100^. All these findings are consistent with previous studies demonstrating the role of QCN nanocarriers in modulating TGF-β signaling during wound healing^101^.

### Histopathological evaluation of QCN-HS in skin

**Fig. 14:**
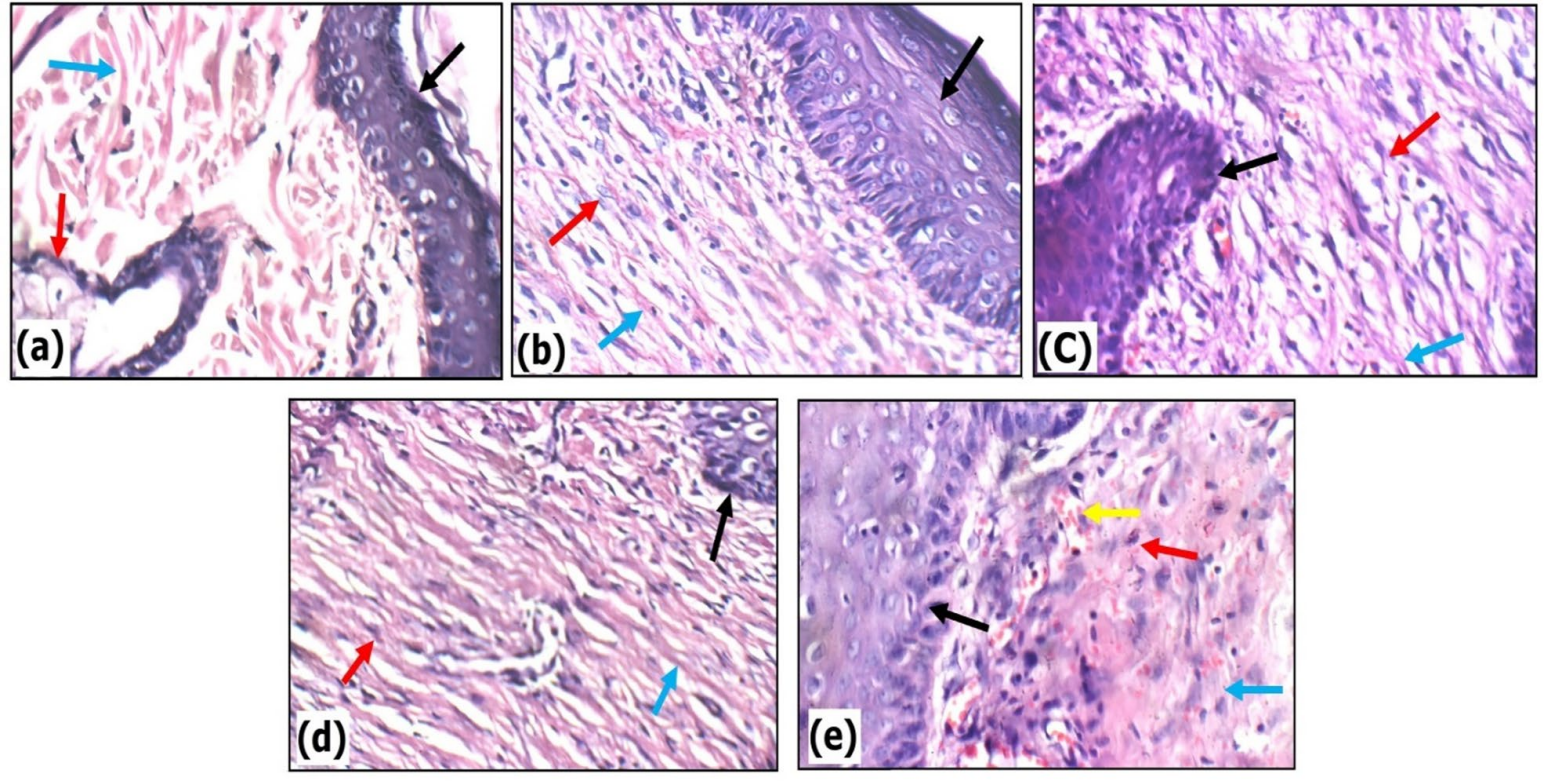
Histopathological evaluation of skin across experimental groups (H&E, X400). (**a**) The Normal Control group shows high power view showing average keratinized epidermis (black arrow), average pilosebaceous units (red arrow), and average collagen (blue arrow), (**b**) The DFU group displays higher power view showing intact epidermis (black arrow), large scar tissue composed of excess active fibroblasts (red arrow), and excess collagen (blue arrow), (**c**) The Fucidin group demonstrates high power view showing intact epidermis (black arrow), small scar tissue composed of inactive fibroblasts (red arrow), and scanty thin collagen (blue arrow), (**d**) The QCN-HS group high power view showing intact epidermis (black arrow), small hypocellular scar tissue composed of few inactive fibroblasts (red arrow) and excess thin collagen (blue arrow), (**e**) The QCN group high power view showing intact epidermis (black arrow), small hypocellular scar tissue composed of few inactive fibroblasts (red arrow), few thin collagen (blue arrow), and extravasated red cells (yellow arrow).

Histological evaluation of skin (Fig. 14) revealed profound pathological alterations in the DFU-GP group, characterized by dense hypercellular scar tissue extending into the subcutis, complete loss of skin adnexa, abundant active fibroblasts, thick disorganized collagen bundles, and widespread inflammatory infiltration accompanied by red blood cell extravasation. Diabetic foot ulcers (DFUs) are marked by chronic tissue damage, with skin exhibiting disrupted architecture, fibroblast hyperplasia, collagen disorganization, and persistent inflammation. These changes reflect a persistent inflammatory state and underscore the need for therapies that promote both local and systemic tissue repair^98^. QCN-GP did not induce significant repair, resulting in disorganized collagen architecture and ongoing inflammatory infiltrates, as QCN’s poor solubility and limited tissue penetration restricted its delivery to the wound microenvironment^65^.

In marked contrast, QCN-HS-GP resulted in the most substantial recovery among all groups, with complete restoration of the keratinized epidermis, minimal hypocellular scar tissue composed predominantly of quiescent fibroblasts, thin and well-organized collagen fibers, and robust neovascularization. The present study demonstrates that QCN-HS nanoformulation exerts pronounced reparative effects across multiple diabetes-compromised tissues. In the skin, QCN-HS-GP effectively reversed hallmark features of chronic diabetic wounds, restoring epidermal continuity, normalizing collagen alignment, and enhancing microvascular density, thus shifting the wound phenotype from non-healing to pro-reparative. These results align with prior studies on nanoformulated Quercetin and its histopathological effect models^102,103^.

### Immunohistochemical evaluation of NF-κB modulation by QCN-HS

**Fig. 15:**
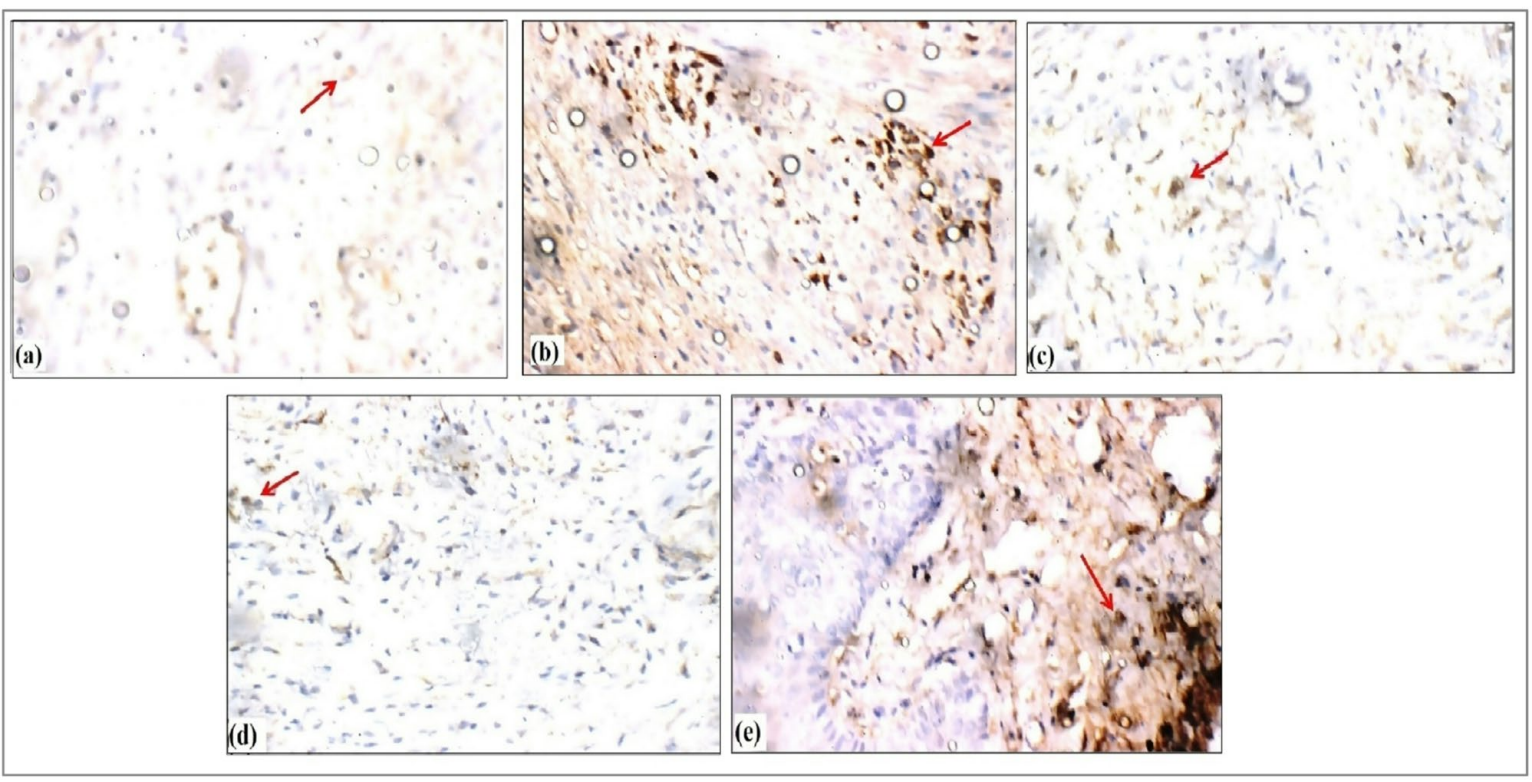
Immunohistochemical expression of NF-κB in skin tissue (Immunostain, X400). (**a**) Normal Control group skin showing negative reactivity (0) for NF-κB (red arrow), (**b**) DFU group skin view showing marked reactivity (+++) for NF-κB (red arrow), (**c**) Fucidin group skin showing mild reactivity (+) for NF-κB (red arrow), (**d**) QCN-HS group skin showing mild reactivity (+) for NF-κB (red arrow), (**e**) QCN group skin showing moderate reactivity (++) for NF-κB (red arrow).

From (Fig. 15), the DFU-GP group exhibited marked and persistent inflammation, as evidenced by strong nuclear localization of NF-κB with intense immunoreactivity (+++). In the diabetic foot ulcer, intense nuclear localization of NF-κB was observed, indicating persistent and unresolved inflammation. This finding aligns with previous reports identifying NF-κB as a major transcription factor, driving the chronic inflammatory response in diabetic wounds. The sustained activation of NF-κB in epidermal and dermal cells reflects an environment of oxidative stress, pro-inflammatory cytokine production, and impaired tissue remodeling, hallmarks of diabetic wound pathology^104^. In contrast, the QCN-HS-GP group demonstrated significantly reduced NF-κB expression, showing only mild reactivity (+), comparable to the negative control (NC-GP), which exhibited no detectable signal (0). QCN-GP displayed moderate NF-κB reactivity (++), indicating partial but incomplete modulation of the inflammatory response. Free Quercetin (QCN) was ineffective in alleviating the NF-κB, as tissues still displayed keratinocyte monodermal dry scalp to epidermal chaos and tissue disorganization. This limited therapeutic efficacy was attributed to QCN’s poor solubility^105^. This attenuation of NF-κB suggests successful suppression of chronic inflammation and a transition toward tissue homeostasis. The QCN-HS also exhibited histological evidence of dermal repair, including reduced inflammatory infiltration, organized collagen deposition, and enhanced vascular density, supporting the observed molecular changes. Moreover, these findings are consistent with previous studies on nanocrystallized flavonoids, which demonstrated that nanoencapsulation enhances cellular uptake, NF-κB- inflammation suppression, and keratinocyte proliferation restoration^106,107^. Notably, our QCN-HS achieved near-complete suppression of NF-κB to control levels, alongside full epidermal restoration, well-aligned collagen, minimal inflammation, and robust vascularization. This dual action targeted anti-inflammatory modulation and complete tissue regeneration demonstrates QCN-HS superior therapeutic potential in chronic diabetic wound healing.

## Conclusion

This study reports the successful development of Quercetin-loaded hyaluosomes (QCN-HS) as a novel platform for diabetic wound management. Integration into a hydrogel matrix enhanced clinical applicability, and cytotoxicity assays verified the safety of Quercetin for topical use. Functionally, QCN-HS gel promoted rapid wound closure in diabetic foot ulcer models and enhanced fibroblast migration in vitro. QCN-HS enhanced the antioxidant defenses and balanced extracellular matrix remodeling. Histopathology confirmed restoration of tissue integrity, and NF-κB downregulation validated anti-inflammatory activity. Collectively, these findings establish QCN-HS gel as a safe and clinically promising nanotherapeutic with strong immunomodulatory and regenerative potential for chronic diabetic wounds.

## Data Availability

The datasets used and/or analysed during the current study are available from the corresponding author on reasonable request.
